# Supplementary material for: Voltage-driven control of single-molecule keto-enol equilibrium in a two-terminal junction system
Source: Nat Commun. 2023 Jun 20;14:3657. doi: 10.1038/s41467-023-39198-7 (PMC10281950; doi:10.1038/s41467-023-39198-7)
Supplement: Supplementary file 1 — Supplementary Information [file 41467_2023_39198_MOESM1_ESM.pdf]

Supplementary Information for

Voltage-driven control of single-molecule keto-enol equilibrium in a two-terminal junction system

Tang et al.

Supplementary Discussions

Synthetic details and characterizations

1,2-Bis(4-(methylthio)phenyl)ethan–1-one **1**: To a solution of 2-(4-(methylthio)phenyl)acetic acid (10 mmol) in 50 mL dichloromethane, thionyl chloride (20 mmol) was added dropwise. A drop of dimethylformamide was also added to the solution. The reaction mixture was stirred for 1 h at room temperature. Then, the solvents were removed under vacuum. 2-(4-(Methylthio)phenyl)acetyl chloride was obtained in quantitative yield without further purification. Thioanisole (13 mmol) was dissolved in 25 mL dichloromethane at 0 ℃, and anhydrous aluminum chloride (13 mmol) was added slowly to the solution. Then, 2-(4-(methylthio)phenyl)acetyl chloride was added to the reaction mixture, and the solution was slowly heated to room temperature. After the (methylthio)phenyl)acetyl chloride was completely consumed, as detected by TLC, the reaction mixture was quenched with ice water. The organic phase was extracted by diethyl ether, which was collected and concentrated. The crude mixture was further purified by column chromatography to give **1**. Yield: 75%. White solid. ^{1}H NMR (500 MHz, $CDCl_3$): δ = 7.90 (dt, *J* = 8.7, 2.0 Hz, 2H), 7.22 (m, 4H), 4.19 (s, –OC*H*$_3$, 3H), 2.51 (s, –SC*H*$_3$, 3H), and 2.46 (s, –SC*H*$_3$, 3H) ppm. ^{13}C NMR (125 MHz, $CDCl_3$): δ = 196.5, 146.1, 136.9, 132.8, 131.5, 129.9, 129.0, 127.1, 125.0, 44.8, 16.0, and 14.7 ppm. HRMS (ESI): *m/z* calcd for $[C_{16}H_{16}NaOS_2]^+$, 311.05348; found, 311.0525.

1,2-Bis(4-(methylthio)phenyl)ethan–1-one–2,2-d$_2$ **1-*d*$_2$**: To a solution of **1** (0.7 mmol) in CD_3OD (5 mL), 0.2 mL NaOD/D_2O solution (10%) was added. The reaction mixture was stirred for 1 h at room temperature. Then, DCl was added dropwise until fully neutralized as detected by pH test strips. The organic phase was extracted by diethyl ether, and the crude mixture was purified by column chromatography to give **1-*d*$_2$**. Yield: 30%. While solid. ^{1}H NMR (500 MHz, $CDCl_3$): δ = 7.90 (dt, *J* = 8.5, 1.8 Hz, 2H), 7.22 (m, 4H), 2.51 (s, –SC*H*$_3$, 3H), and 2.46 (s, –SC*H*$_3$, 3H) ppm. HRMS (ESI): *m/z* calcd for $[C_{16}H_{14}\ D_2NaOS_2]^+$, 313.06603; found, 313.0652.

(Z)-((1-methoxyethene–1,2-diyl)bis(4,1-phenylene))bis(methylsulfane) **2-OMe**: To a solution of **1** (0.7 mmol) in methanol (10 mL), trimethyl ester (0.85 mmol) and 4-toluenesulfonic acid (0.1 mmol) were added. The reaction mixture was stirred at 60 °C for 12 h. Then, the reaction was quenched by an ammonium chloride solution. The crude mixture was extracted from diethyl ether and purified by column chromatography to give **2-OMe**. Yield: 35%. White solid. ^{1}H NMR (400 MHz, $CDCl_3$): δ = 7.63 (d, *J* = 8.2 Hz, 2H), 7.47 (d, *J* = 8.2 Hz, 2H), 7.25 (m, 4H), 6.06 (s, 1H), 3.63 (s, –OC*H*$_3$, 3H), 2.52 (s, –SC*H*$_3$, 3H), and 2.50 (s, –SC*H*$_3$, 3H) ppm. ^{13}C NMR (100 MHz, $CDCl_3$): δ = 155.6, 138.9, 136.4, 133.0, 132.9, 129.0, 126.8, 126.8, 126.6, 126.3, 111.9, 57.9, 15.9, and 15.6 ppm.

Plotting the dual distributions in Fig. 2d.

We first separate the total data into two groups based on the measured plateau lengths between $10^{-3.2}$ to $10^{-1.1}$ G_0. Taking the data in 0.5 V bias as an example, as shown in Supplementary Fig. 25a and Supplementary Fig. 25c, we observe two patterns of 1D conductance histograms, which are corresponding to the high and low conductance states, respectively. The corresponding 2D conductance histograms are shown in Supplementary Fig. 25b and Supplementary Fig. 25d. We plotted the two separated groups together with the total data in the same 1D conductance histogram. As shown in Fig. 2d, the total data is shown in the blue histograms. The blue and red curves are the profiles of the histograms of the two separated groups. We then used Gaussian fitting to fit the conductance peaks in blue and red curves, leading to the corresponding blue and red areas.

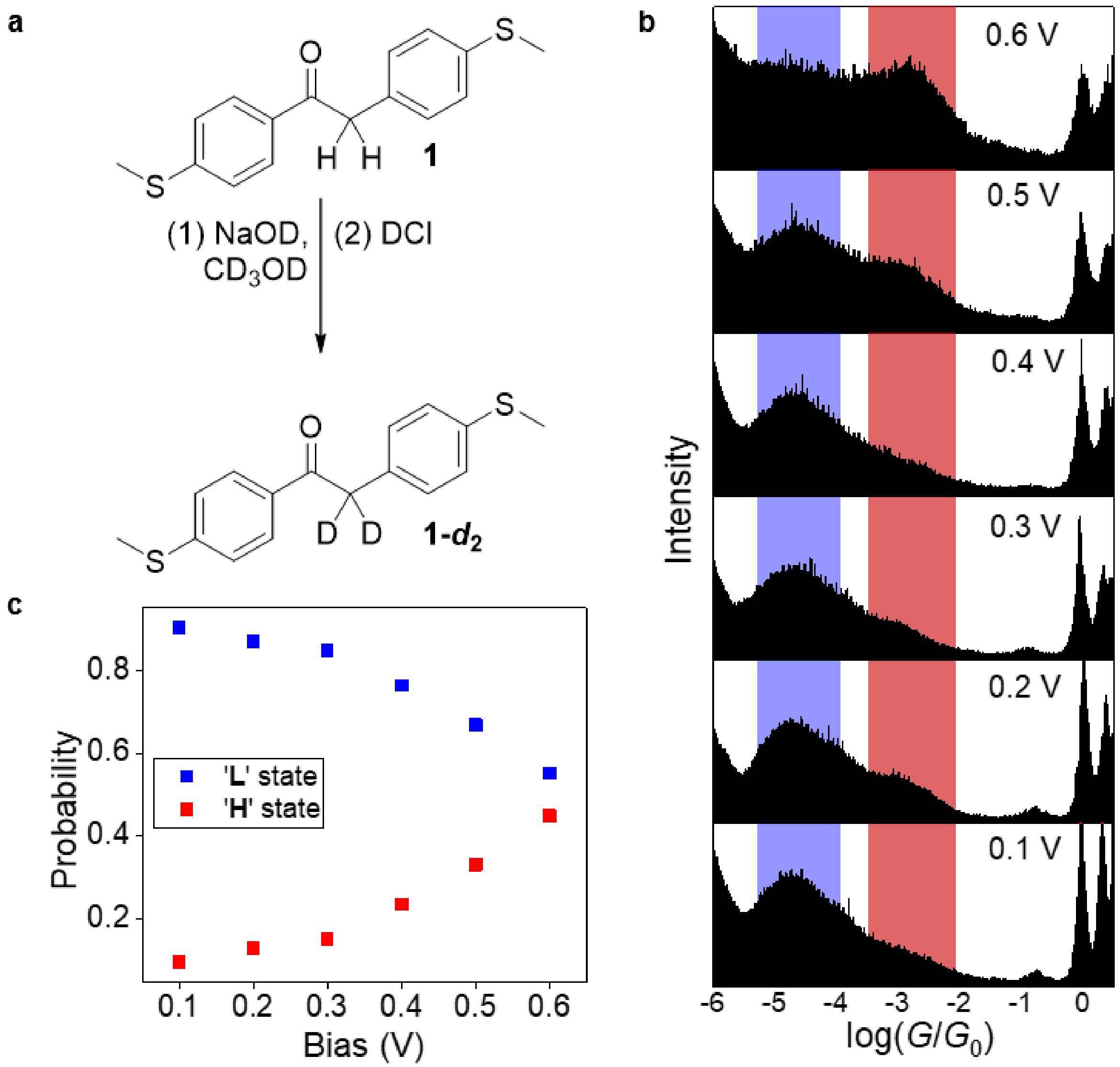

Supplementary Figure 1. 1D conductance histograms for the deuterated analogue 1-d_2. a 1-d_2 is synthesized by adding NaOD into a CD_3OD solution of **1** (1), which is further neutralized by DCl (2). **b** 1D conductance histograms of **1-d_2** at different biases. The STM-BJ experiments are performed on the 0.1 mM solution of **1-d_2** in TCB under ambient conditions. **c** Distribution probabilities of states 'L' and 'H' are plotted against different biases. The emergence of the 'H' peak in the conductance histograms is not altered for deuterated **1-d_2** compared to the regular **1** (cf. Fig. 2), indicating that proton tunneling is not a significant factor in the tautomerization mechanism. The 'H' state of **1-d_2** shows a 4% lower distribution than that of **1** at 0.6 V.

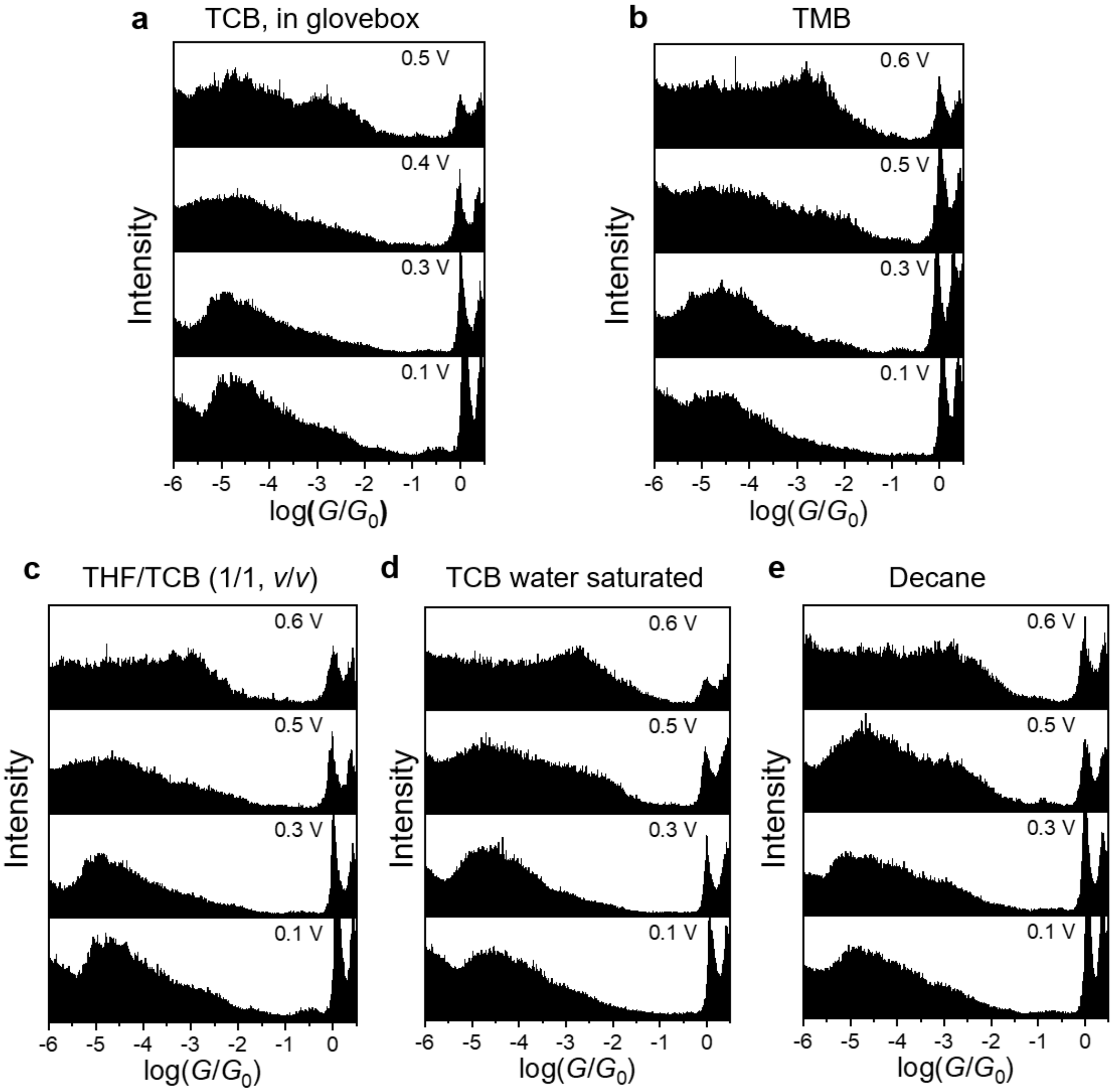

Supplementary Figure 2. 1D conductance histograms of the water control experiments. a The experiment was performed in a glovebox and a dry TCB solvent. **b–e** The following STM-BJ experiments were performed in the corresponding solvents under the conditions shown above the 1D conductance histograms. The control experiments (**b–e**) were performed under ambient conditions. TMB is the abbreviation for sym-trimethylbenzene (**b**). All STM-BJ experiments were performed on a 0.1 mM solution of **1.** The THF we used has an initial moisture content of ~2%, which will lead to higher moisture in the solvent mixture of THF/TCB (**c**) than TCB itself. The water-saturated TCB was prepared by vigorously mixing with an equivalent volume of water and TCB in a glass vial. Then the water/TCB mixture was stood for 24h to get phase separation. The TCB phase, i.e., the water-saturated TCB, was carefully taken out by pipette for the following conductance experiment (**d**). The experiment in decane showed the same tendency (**e**). The emergence of the 'H' peak in the conductance histograms was not significantly affected by the presence/absence of water. Therefore, we think water may not significantly accelerate or inhibit the tautomerization process in the STM-BJ experiments.

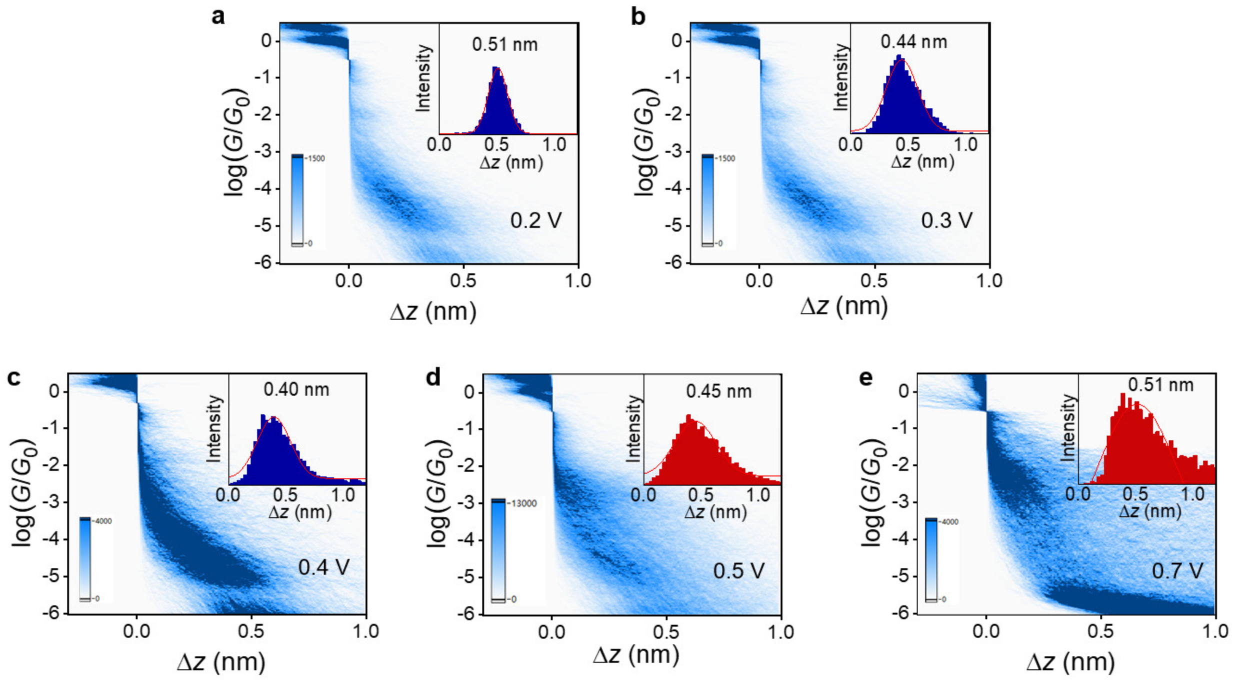

Supplementary Figure 3. Two-dimensional conductance histograms. a–e Two-dimensional conductance histograms of compound **1** at different biases, 0.2 V (**a**), 0.3 V (**b**), 0.4 V (**c**), 0.5 V (**d**), and 0.7 V (**e**). The stretching distances (Δ*z*) are shown in the inset, and the Gaussian fitting determines the peak centers. There is a 0.50 nm snap-back distance after the breaking of the gold-gold contact. For example, the corrected stretching distance at a 0.2 V bias is 0.94 nm (0.44 + 0.50 nm).

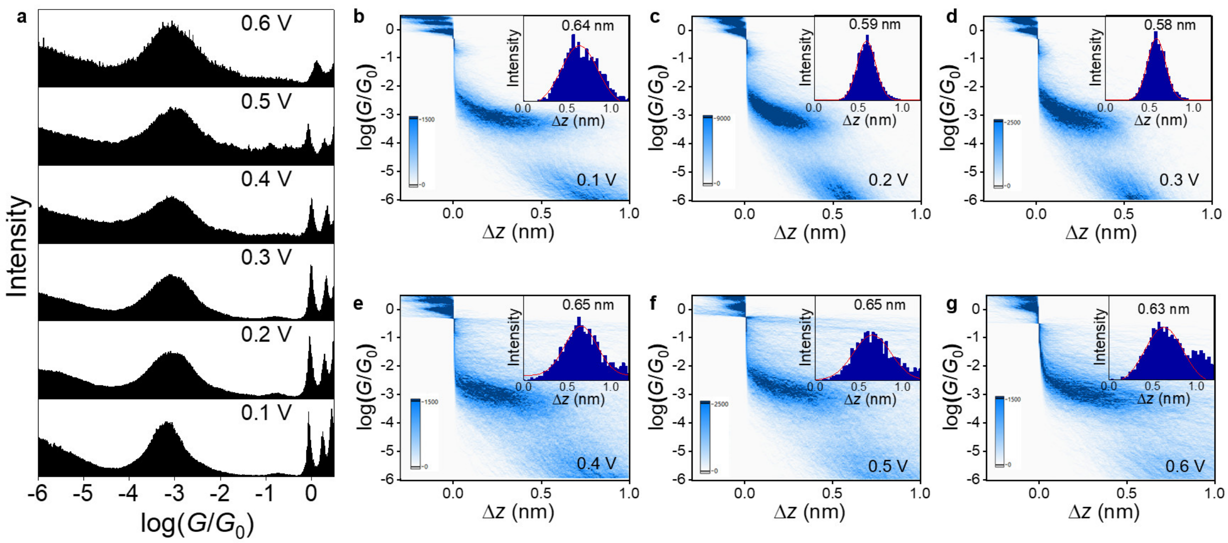

Supplementary Figure 4. Control experiments on the methylated compound 2-OMe. a–g 1D (**a**) and 2D (**b–g**) conductance histograms of **2-OMe**. All the STM-BJ experiments were performed on a 0.1 mM solution of **2-OMe** within different biases 0.1 (**b**), 0.2 (**c**), 0.3 (**d**), 0.4 (**e**), 0.5 (**f**), and 0.6 V (**g**). The insets show the corresponding distribution of stretching distance.

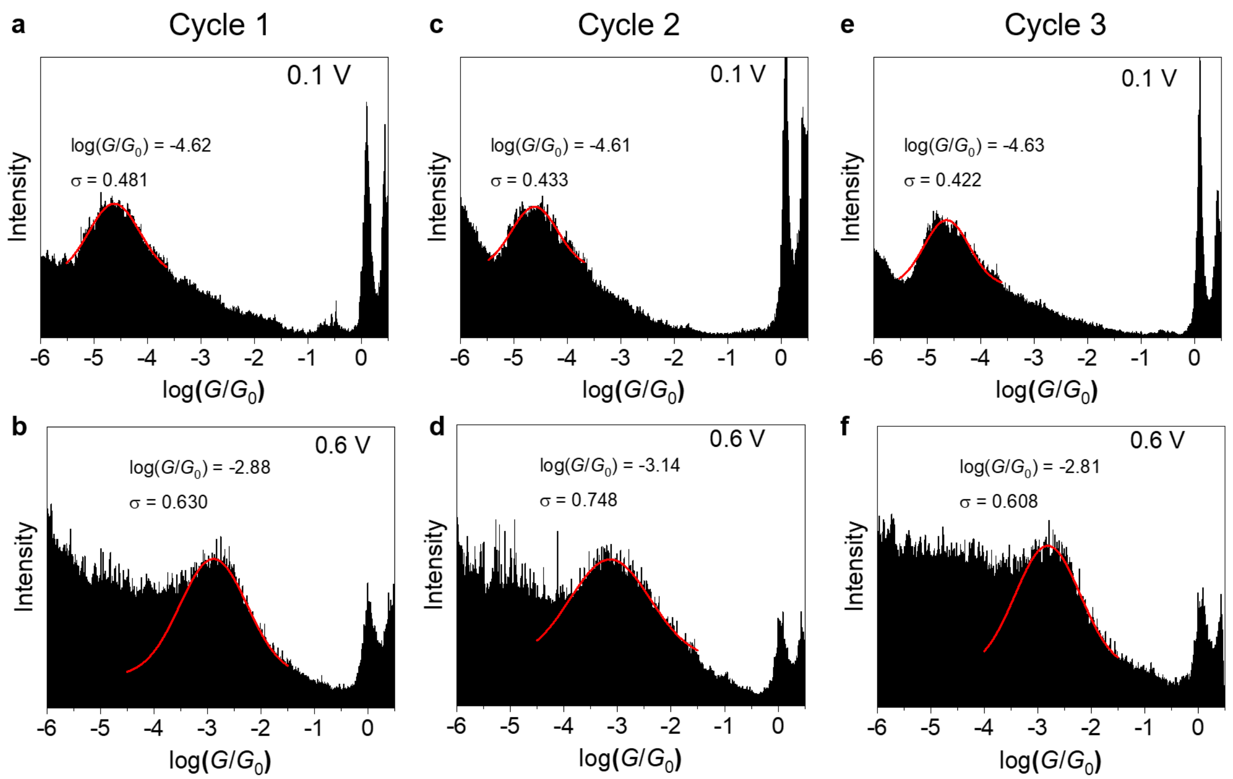

Supplementary Figure 5. Switching experiments. a–f 1D conductance histograms of compound **1** with bias switching between 0.1 and 0.6 V in cycles 1 (**a–b**), 2 (**c–d**), and 3 (**e–f**). Peak centers of the dominant conductance peaks are determined by the Gaussian fitting in the corresponding 1D conductance histograms. The error was defined as the standard deviation of the Gaussian fitting.

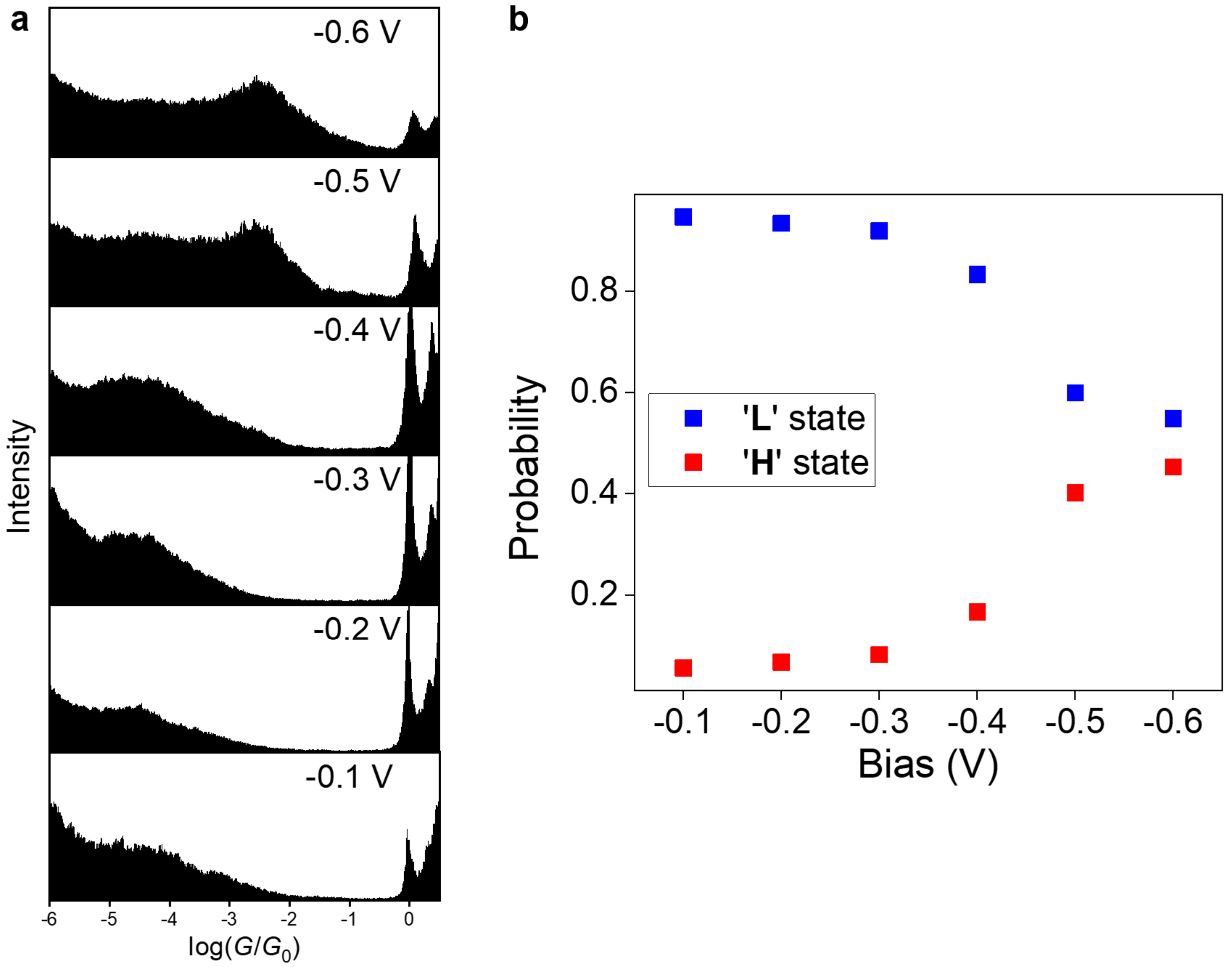

Supplementary Figure 6. Control experiments in negative bias. a 1D conductance histograms of compound **1** were obtained with different biases. The STM-BJ experiments are performed on the 0.1 mM solution of compound **1** in TCB under ambient conditions. **b** The distribution probabilities of states 'L' and 'H' are plotted against different biases.

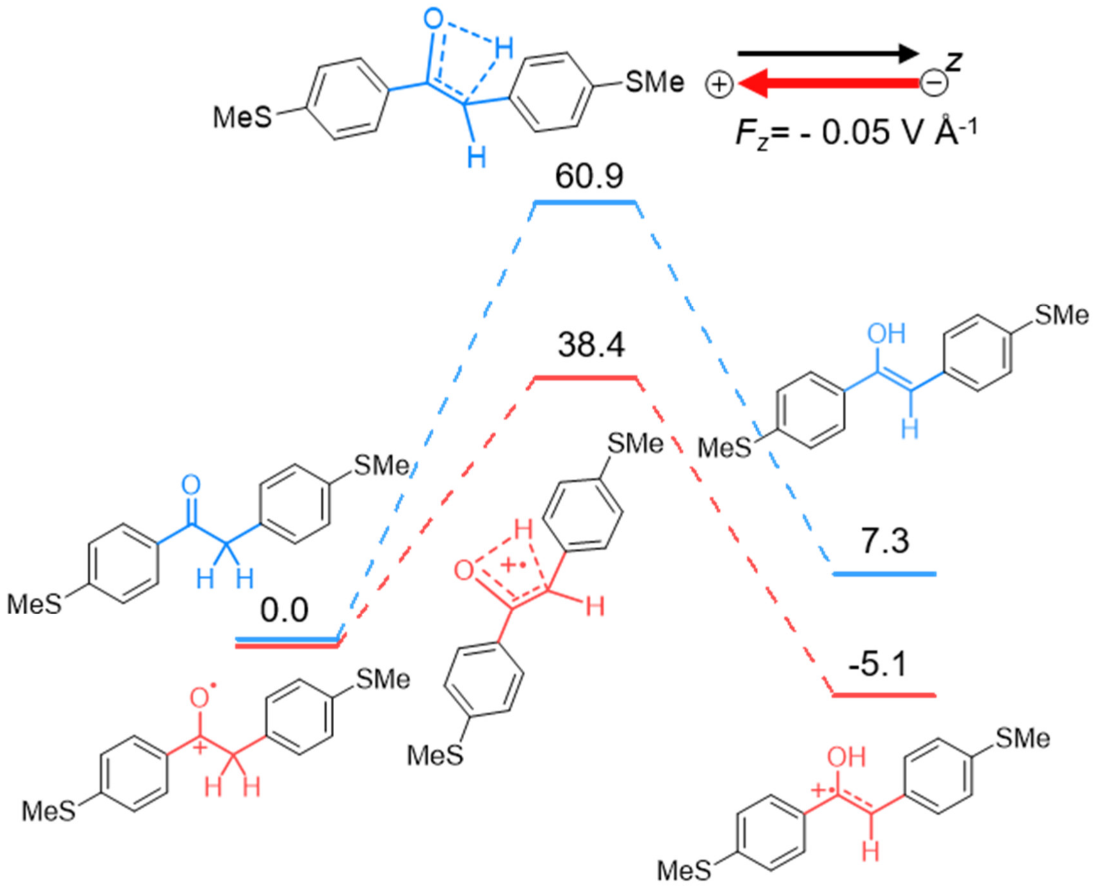

Supplementary Figure 7. PES in the electric field. Reaction profile (in kcal mol^{-1}) associated with the tautomerization reaction on the ground-state, i.e., uncharged, PES (blue), and on the PES of the radical, cationic species obtained after charge injection (red), calculated at B3LYP/def2-TZVP level of theory in the case of $F_Z = -0.05$ V Å^{-1}. Energies are denoted in kcal mol^{-1}. The blue and red curves represent low- and high-conductance states.

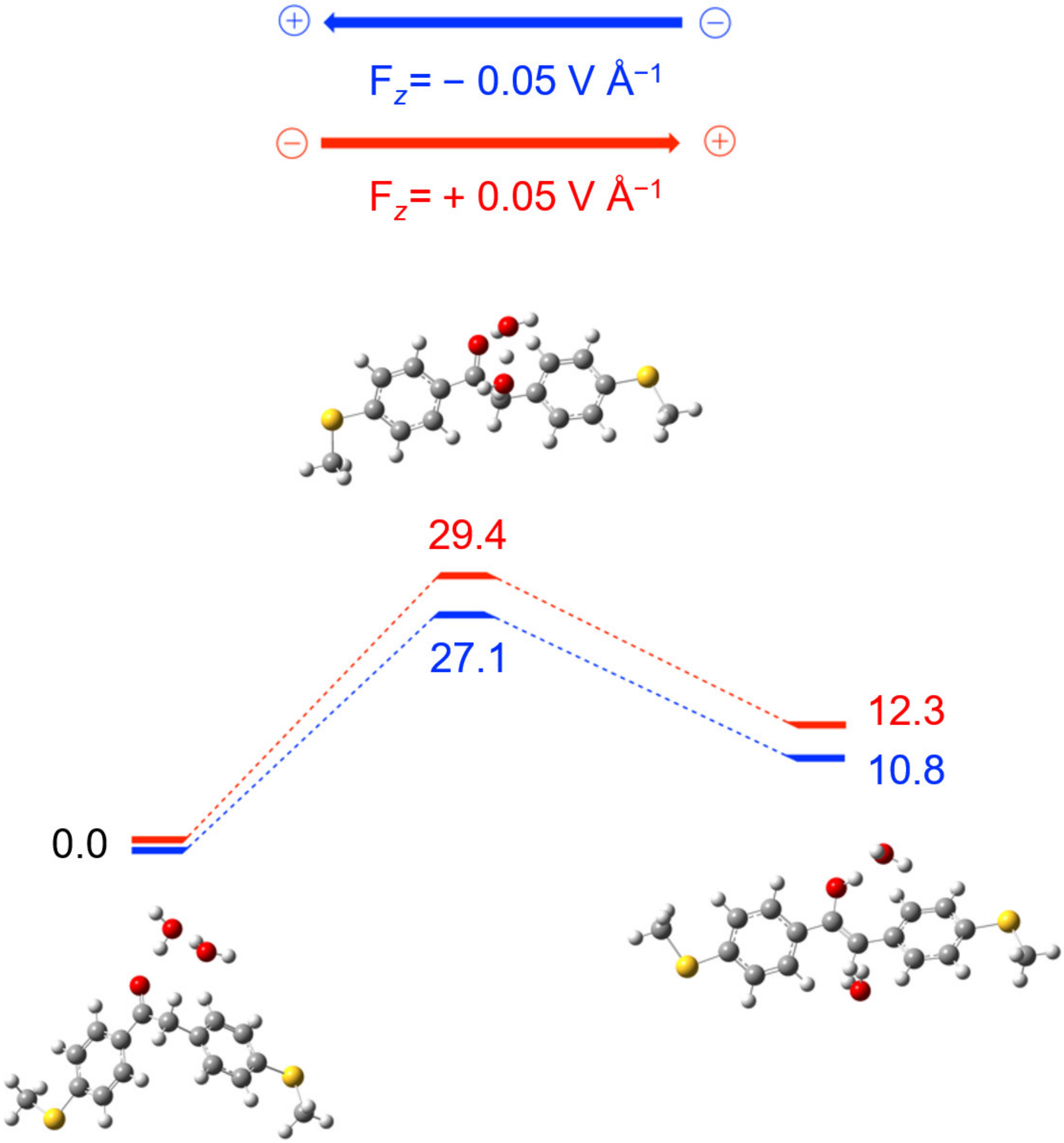

Supplementary Figure 8. H_2O-assisted PES. Potential energy profiles associated with the rejected alternative H_2O-assisted reaction mechanism for the uncharged species (**1**) in the cases of $F_Z = -0.05$ V Å^{-1} (blue) and $F_Z = +0.05$ V Å^{-1} (red). Energies are denoted in kcal mol^{-1}.

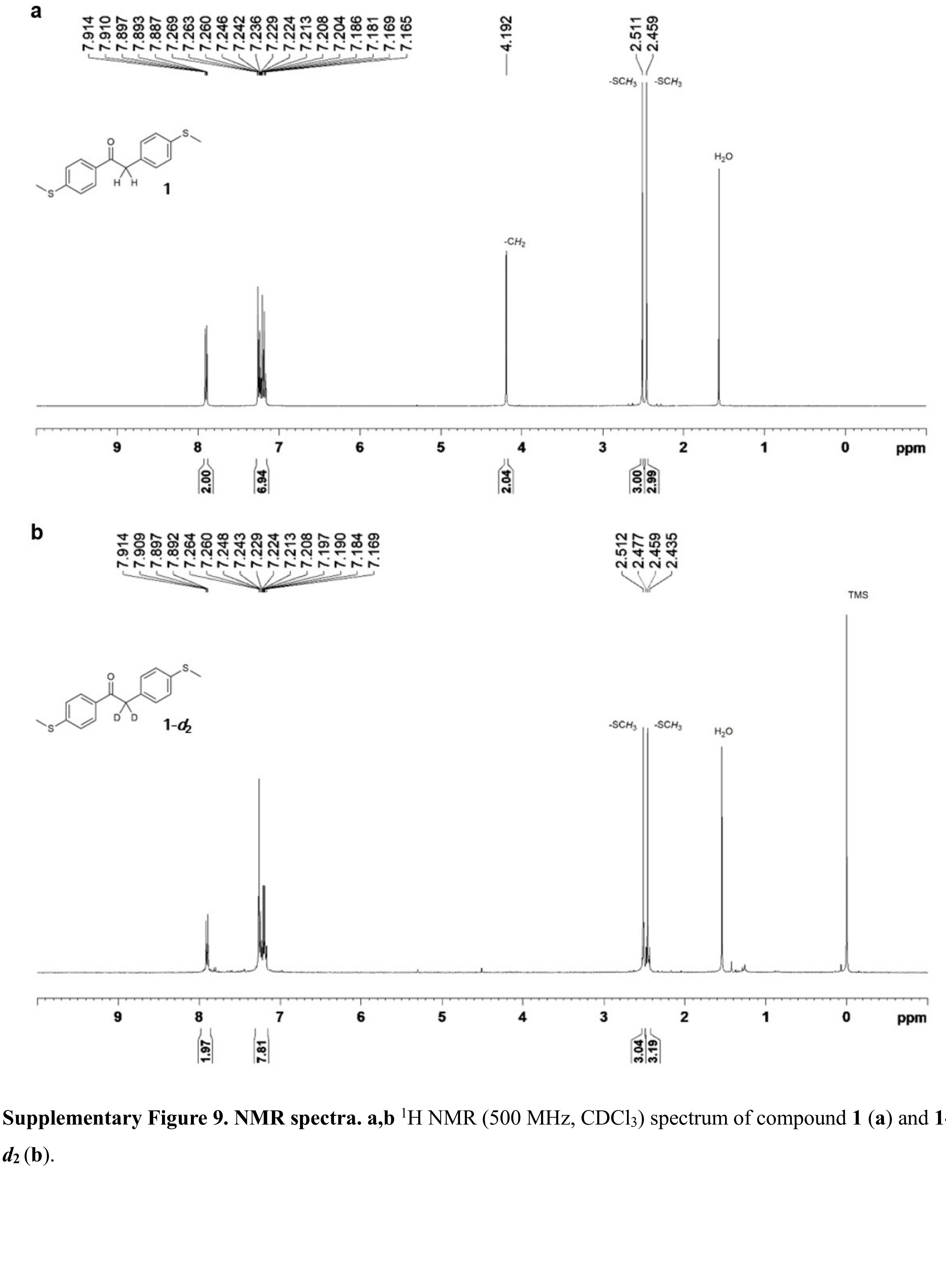

Supplementary Figure 9. NMR spectra. a,b ^{1}H NMR (500 MHz, $CDCl_3$) spectrum of compound **1** (**a**) and **1-*d*$_2$** (**b**).

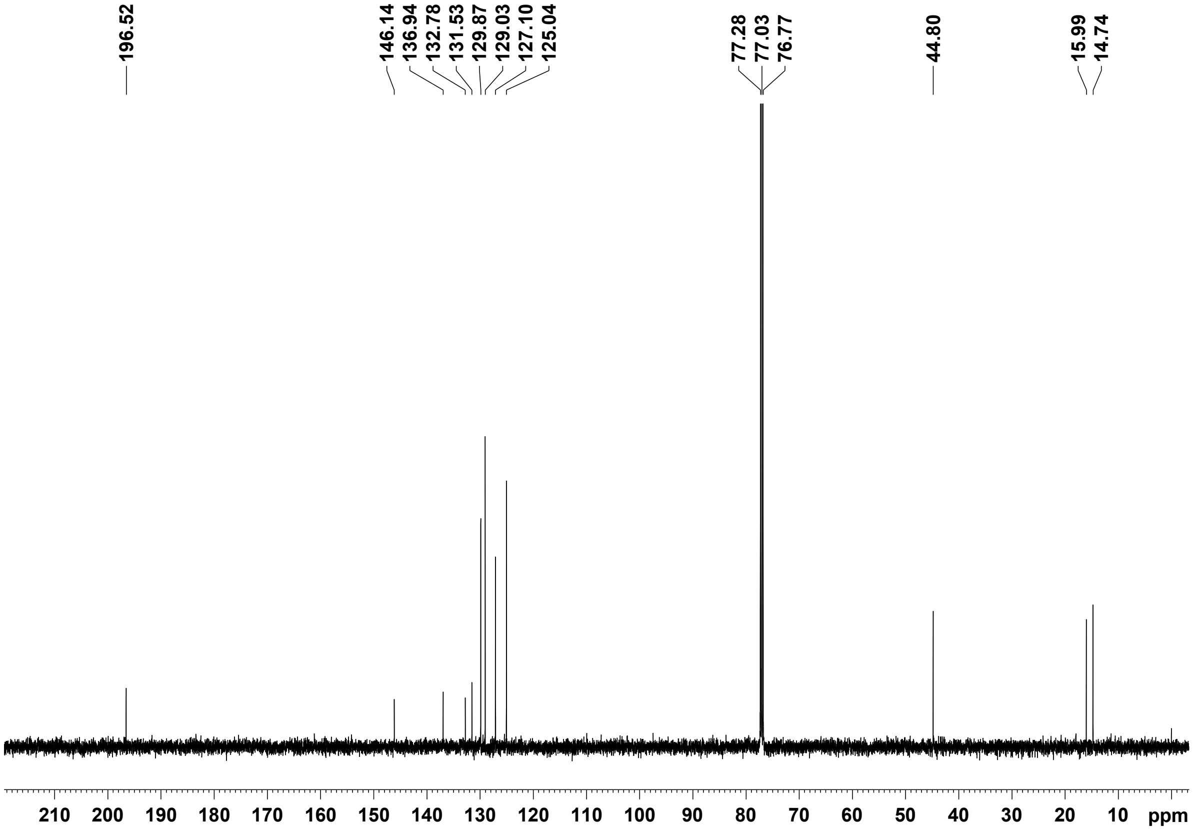

Supplementary Figure 10. NMR spectra. The ^{13}C NMR (125 MHz, $CDCl_3$) spectrum of compound **1**.

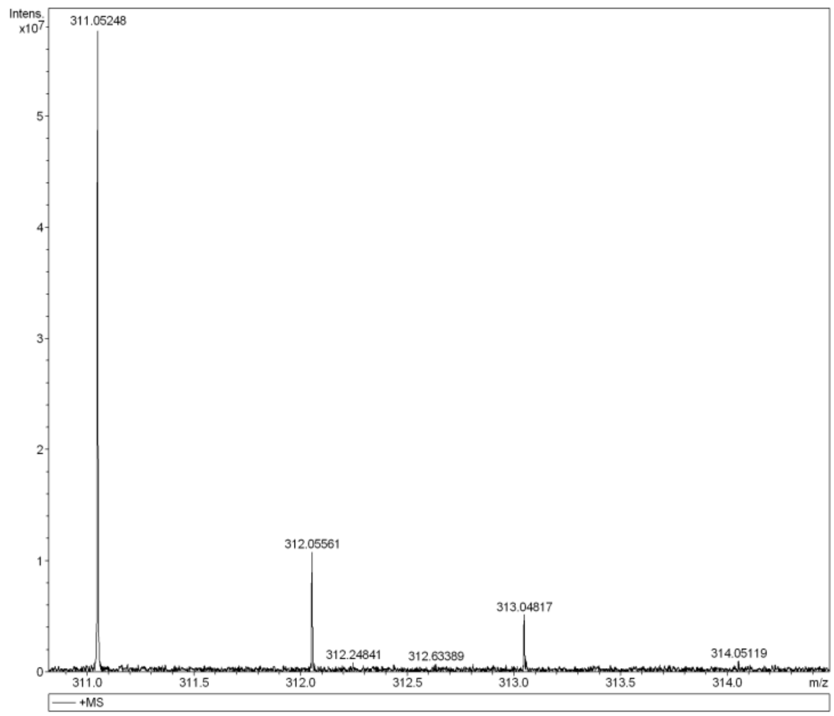

Supplementary Figure 11. MS spectra. Positive-ion ESI-MS spectrum of [**1**+Na+] $[C_{16}H_{16}NaOS_2]^+$ measured in methanol. The theoretical mass is 311.05348.

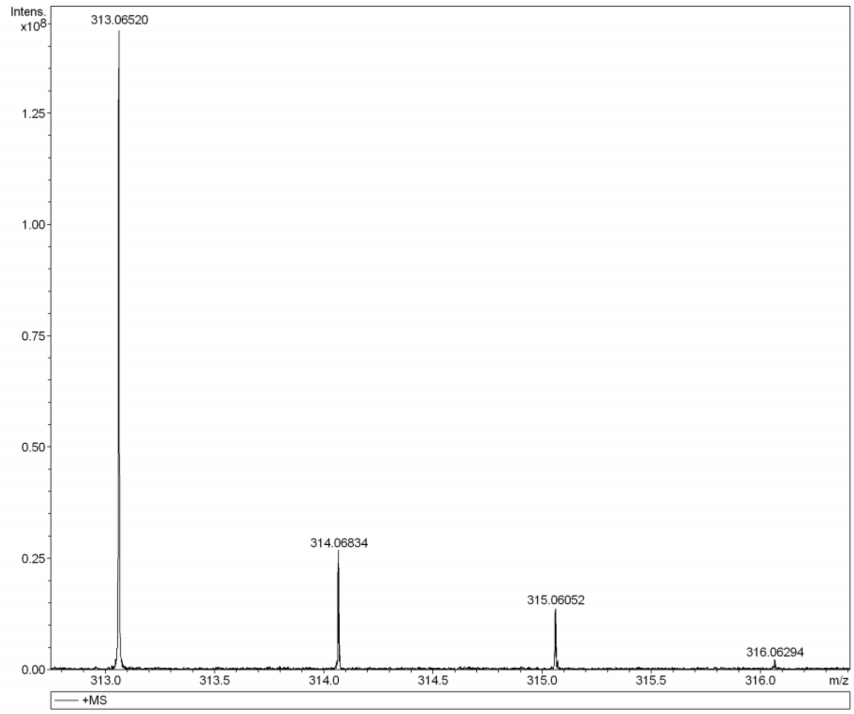

Supplementary Figure 12. MS spectra. Positive-ion ESI-MS spectrum of [**1-*d*₂**+Na$^+$] $[C_{16}H_{14}D_2NaOS_2]^+$ measured in methanol. The theoretical mass is 313.06603.

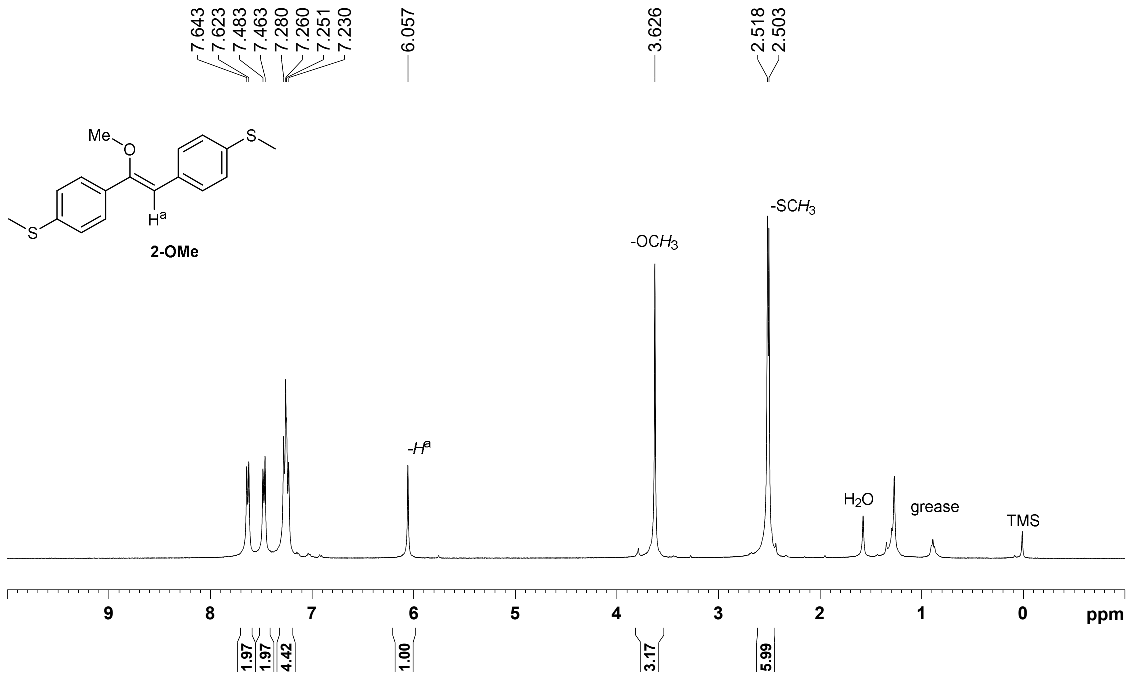

Supplementary Figure 13. NMR spectra. The ^{1}H NMR (400 MHz, $CDCl_3$) spectrum of compound **2-OMe**.

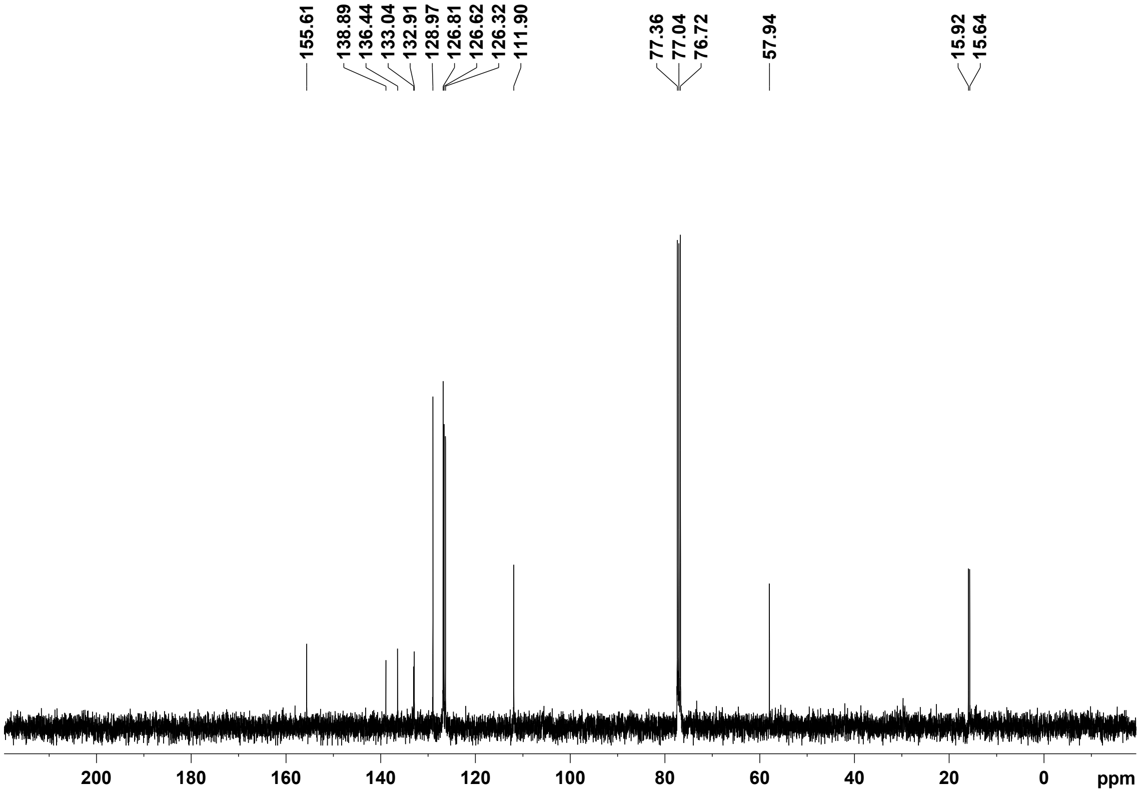

Supplementary Figure 14. NMR spectra. The ^{13}C NMR (100 MHz, $CDCl_3$) spectrum of compound **2-OMe**.

Supplementary Note 1. Control experiments.

Proving the charge injection mechanism by EC-STMBJ experiments. We have done an electrochemical (EC) STM-BJ experiment in a four-electrode system[1] to oxidize the molecule in-situ and simultaneously measure the corresponding single-molecule conductance (Supplementary Fig. 15). The ECSTM-BJ was performed with 1.0 mM molecule **1** and 0.1 M tetrabutylammonium hexafluorophosphate as the electrolyte. The working, reference, and counter electrodes are the gold tip, Ag/AgCl, and platinum wire. In the conductance characterization, the gold tip was coated with Apiezon wax[2] to reduce background capacitive current and electrochemical currents.

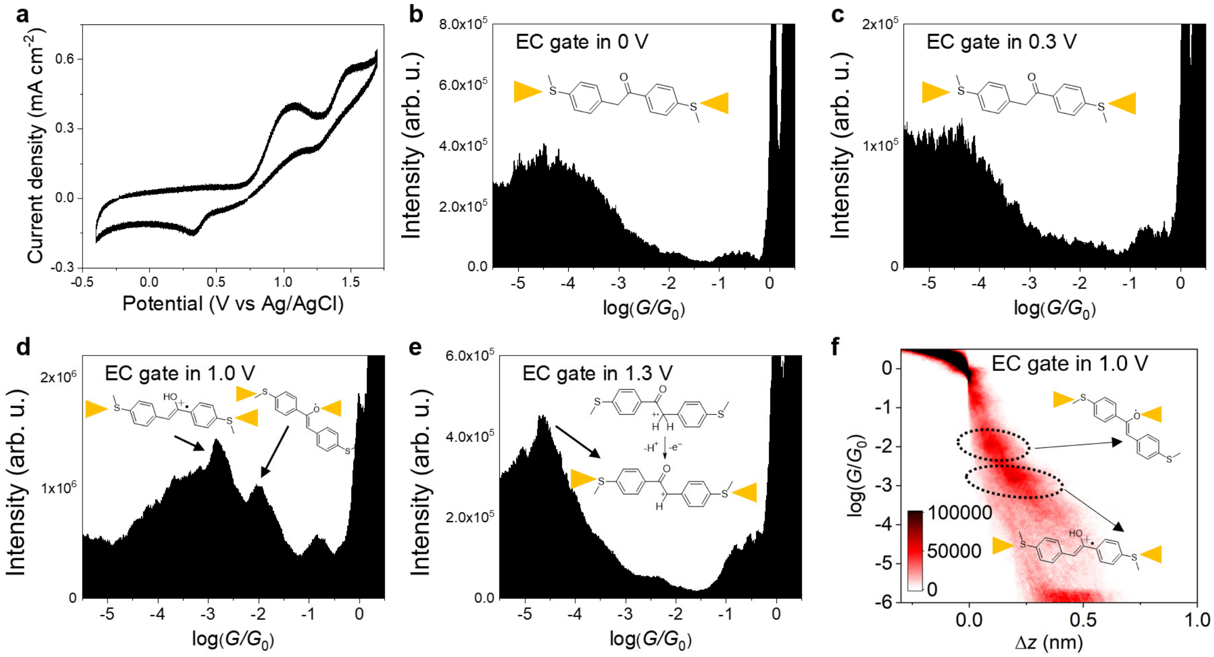

Supplementary Figure 15. The electrochemical STM-BJ experiments for compound 1. a The cyclic voltammetry is characterized in the propylene carbonate with 1.0 mM molecule **1** and 0.1 M tetrabutylammonium hexafluorophosphate as the electrolyte. (**b-f**) The 1D conductance histograms in 0 (**b**), 0.3 (**c**), 1.0 (**d**), and 1.3 V (**e**) EC gates, respectively. All the above conductance measurements are performed under ambient conditions with 0.1 V bias applied. (**f**) The 2D conductance histograms in 1.0 V EC gate.

As shown in Supplementary Fig. 15a, the cyclic voltammetry characterization was started at −0.2 V with an oxidative scan by the ECSTM-BJ setup. In the oxidative scan, the cyclic voltammetry of molecule **1** shows two consecutive oxidation peaks at around 1.0 V and 1.3 V EC potentials (relative to Ag/AgCl), which correspond to the one-electron and two-electron oxidization and suggest that oxidation states are accessible. We observed three reduction peaks at around 0.3, 0.7, and 1.2 V in the reductive scan. The reductive peaks at 0.7 and 1.2 V should be the redox pairs corresponding to oxidation peaks at 1.0 and 1.3 V. The new reductive peak that

appeared in the reductive scan at 0.3 V suggests that there was a significant structural reorganization in the oxidative scan.

We use an EC gate and measure the single-molecule conductance simultaneously. All the conductance characterizations are performed with 0.1 V bias applied between the tip and substrate. As shown in Supplementary Fig. 15b, the 0 V EC gate measurement reveals a mono-conductance state around $10^{-4.5}$ G_0, which is the low-conductance state (keto form) of molecule **1**. At the 0.3 V EC gate (Supplementary Fig. 15c), there was only the signal of the low-conductance state. When applying a 1.0 V EC gate, we observe two distinctive conductance peaks, centering around $10^{-2.0}$ G_0 and $10^{-2.8}$ G_0 (Supplementary Fig. 15d). Its 2D conductance histogram indicates that the conductance plateaus of $10^{-2.8}$ G_0 are about two times longer than the plateaus of $10^{-2.0}$ G_0 (shown in Supplementary Fig. 15f), while the two types of plateaus occur consecutively. The conductance plateaus of $10^{-2.8}$ G_0 are very similar to the high-conductance state (enol form) of the two-electrode measurement at 0.6 V (Fig. 2d). We also observe that upon two-electron oxidation, a low-conductance state is reached again (shown in Supplementary Fig. 15e). This state most likely corresponds to a deprotonated keto form.

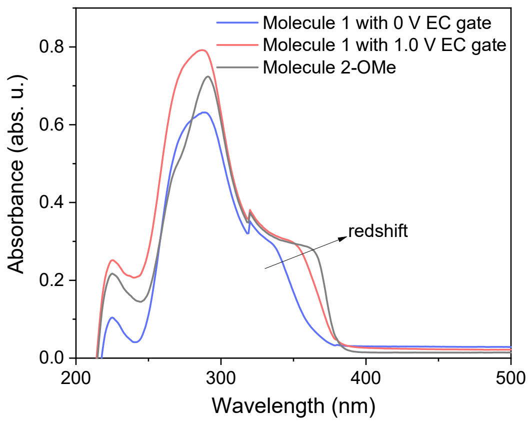

Supplementary Figure 16. The UV/Vis spectra for the electrochemical product. The UV/Vis spectra of **1** in 0 V and 1.0 V EC gate, with the molecule **2-OMe** as reference. All the UV/Vis spectra are characterized with 0.1 mM target molecules in a solution of propylene carbonate with 0.1 M tetrabutylammonium hexafluorophosphate added.

To understand the structure in oxidation, we also measured the UV/Vis spectra of the oxidized species. We expect that the enol form will have a more extended pi-system than that of the corresponding keto form. Therefore, the enol form will have a smaller bandgap than that of the keto form. As expected, the methylated enol form **1-OMe** (with the extended π-system) exhibits a redshift absorption (50 nm) with respect to molecule **1** (Supplementary Fig. 16, grey curve). More importantly, after one one-electron oxidation in molecule **1**, we observed a similar redshift absorption (Supplementary Fig. 16, red curve), which is consistent with the absorption of **1-OMe**. The UV/Vis spectra further support that the enol form was generated from the keto form upon one-

electron oxidation, and provide additional proof that the high-conductance state indeed corresponds to an enol structure.

Excluding the possibility of connectivity change. To exclude the possibility that conductance switching comes from connectivity change, we perform a series of control experiments. As shown in Supplementary Fig. 17a, we synthesized reference molecules **R1** and **R2** with one –SMe anchor removed compared to molecule **1**. Subsequently, we characterize the single-molecule conductance of these molecules by STM-BJ. As shown in Supplementary Fig. 17b and 17c, the 1D conductance histograms do not show any clear conductance peak with the bias ranging from 0.1 V to 0.6 V, which is in sharp contrast to the result of molecule **1** shown in Fig. 2d. The above results suggest that the oxygen in molecule **1** cannot contact gold electrodes to form stable and observable junctions even with a higher bias applied. If the high-conductance state were originating from a shorter junction geometry, we would expect to observe two consecutive conductance plateaus, suggesting a positive correlation between the high- and low-conductance states. To examine whether such a correlation exists, we performed a correlation analysis. As shown in Supplementary Fig. 28b, the correlation matrix for the dataset of 0.6 V bias shows a clear negative correlation (red areas) between the high- and low-conductance values, which means that the two conductance plateaus occurred in a mutually exclusive pattern. As a reference, such a negative correlation does not emerge from the dataset at 0.1 V bias (Supplementary Fig. 28a).

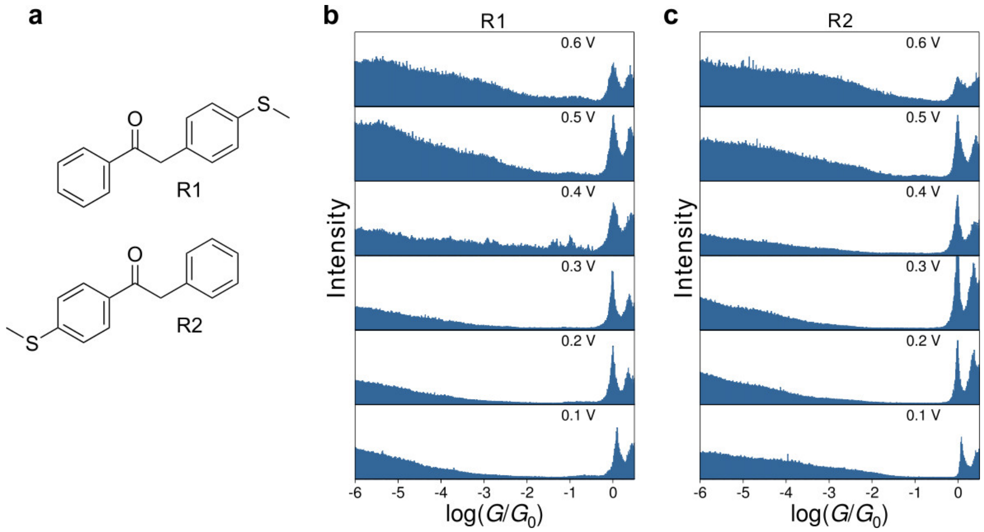

Supplementary Figure 17. Control experiments for molecules with one –SMe anchor. a The chemical structures of reference molecules R1 and R2. **b,c** The 1D conductance histograms of R1 (**b**) and R2 (**c**) in the bias ranging from 0.1 to 0.6 V. The above experiments are performed with 0.1 mM target molecules in the 1,2,4-trichlorobenzene solvent at room temperature.

Tip-speed related state switching probability. As indicated in the manuscript, tautomerization involves a non-negligible reaction barrier that needs to be crossed, and consequently, the switching takes some time to transpire.

As a result, the tip speed can be expected to affect the ratio of the high- and low-conductance states. We performed some additional experiments to confirm this reasoning. As shown in Supplementary Fig. 18a, when we change the tip speeds in the STM-BJ experiments with 0.5 V bias applied, the ratio of high-conductance states indeed decreases significantly as the tip speed is increased from 5 to 20 nm s^{-1}. In the 2D conductance histograms, the high-conductance plateaus in Supplementary Fig. 18b are much more significant than in Supplementary Fig. 18c. We are considering that there is about a 0.5 nm stretching distance before the contact of a molecular junction breaks down. Thus, we could estimate the junction lifetime by dividing the stretching distance by the tip speeds. As shown in Supplementary Fig. 18d, we find that the high-conductance state ratio is increased with the longer junction lifetime. The above results indicate that the amount of transport charge is also vital for the induced tautomerization.

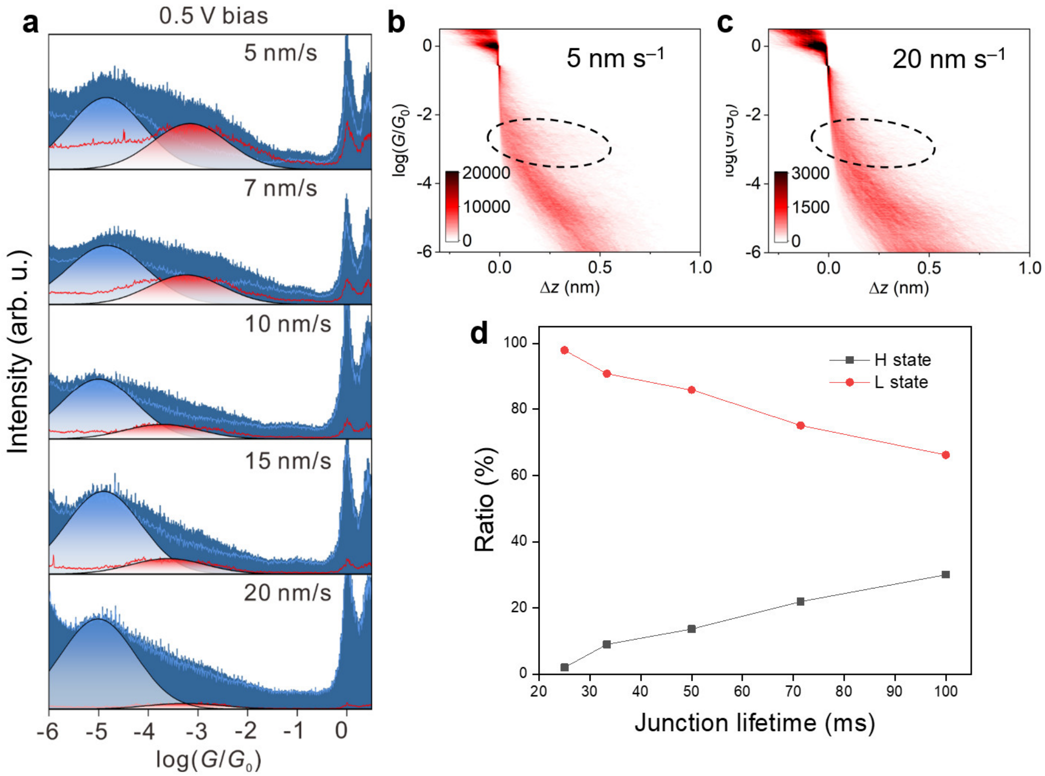

Supplementary Figure 18. The STM-BJ measurement of 1 at different tip speeds. a The 1D conductance histograms at different tip speeds. The blue and red lines represent the 1D conductance histograms of low- and high-conductance states with the corresponding Gaussian fitting shown in blue and red areas. The dark blue histograms are plotted from all the data without selection. **b**–**c** The 2D conductance histograms for the measurements at 5 nm s^{-1} (**b**) and 20 nm s^{-1} (**c**) tip speed. The color scales represent intensity. The dashed cycle represents the region of the high-conductance state. **d** The ratio of low- and high-conductance states are plotted against the corresponding junction lifetime.

Supplementary Note 2. Valence Bond interpretation of the impact of oxidation on the PES.

In this Supplementary note, we will take a closer look at the root cause for the dramatic difference in tautomerization behavior of the uncharged and charged species of the molecular bridge. The electron transfer-triggered switch in thermodynamic preference can be straightforwardly understood by simply considering some common bond dissociation enthalpy (BDE) tables found in the literature[3]. To simplify the analysis and facilitate the discussion, we start by considering the tautomeric system in the absence of thiol-linkers. The potential energy profiles associated with tautomerization (with opposing electric field directions, cf. the main text) for this system are presented in Supplementary Fig. 19.

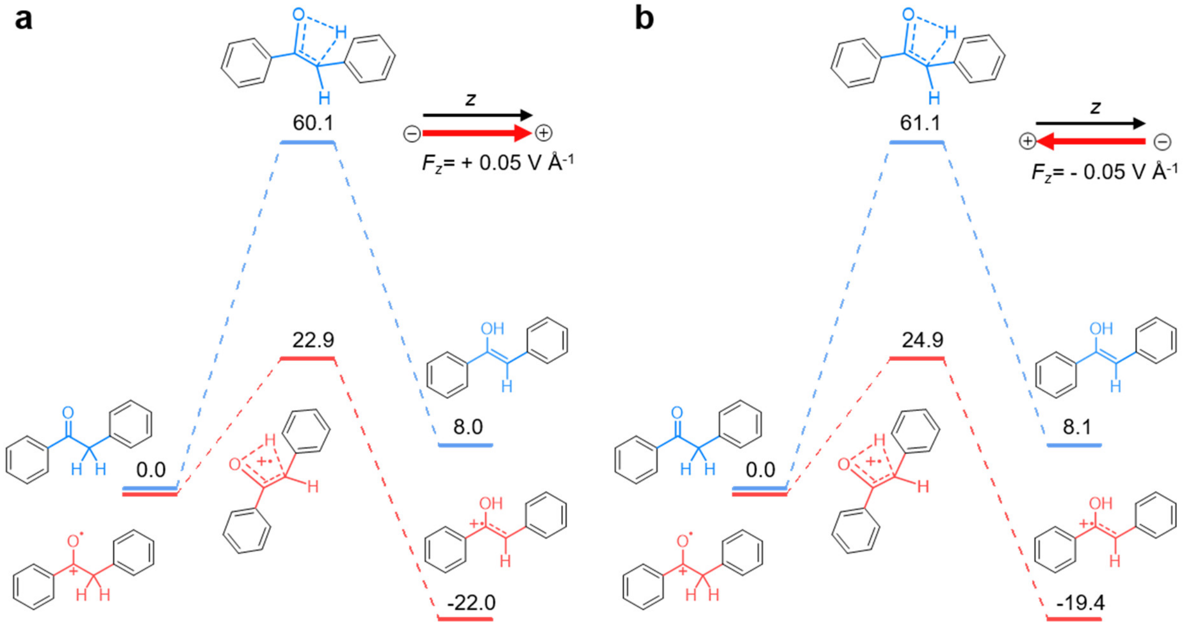

Supplementary Figure 19. PESs of reference molecules without –SMe groups. a Reaction profile (in kcal mol^{-1}) associated with the tautomerization reaction on the ground-state, i.e., uncharged, PES (blue), and on the PES of the radical, cationic species obtained after charge injection (red), calculated at B3LYP/def2-TZVP level of theory with an electric field $F_Z = +0.05$ V Å^{-1}. **b** The same reaction profile, but then with $F_Z = -0.05$ V Å^{-1}.

Supplementary Fig. 19 clearly reveals that in the case of the uncharged species, the reactant bonds broken throughout the reaction are an RR'C=O (BDE ≈ 98 kcal mol^{-1}) and HRR'C–H (BDE ≈ 105 kcal mol^{-1}) bond, and the product bonds being formed are the RO-H (BDE ≈ 110 kcal mol^{-1}) and RR'C=CHR" (BDE ≈ 85 kcal mol^{-1}) bond (Supplementary Fig. 20a). As such, the thermodynamic driving force ΔE_{rp} associated with this reaction can be estimated at +8 kcal mol^{-1} (i.e., 98 + 105 – 110 – 85). Note that this crude estimate agrees perfectly with the calculated value presented in Supplementary Fig. 19.

In the case of charged species, it is straightforward to see that – in the idealized situation that the electron is removed exclusively from the keto-/enol-moiety of the molecule under consideration – there is only a single active bond in the tautomerization process: the HRR'C–H bond is broken in the reactant and replaced by the RO–H one in the product (Supplementary Fig. 20b). Using the same bond dissociation enthalpy values as for the uncharged species,[1] one ends up with an estimated thermodynamic driving force ΔE_{rp} of −5 kcal mol^{-1}. As such, we readily recover the experimentally observed thermodynamic switching, i.e., ΔE_{rp} goes from $+8 \rightarrow -5$ kcal mol^{-1} upon removing an electron from the system. However, it should be clear that the obtained driving force does not correspond numerically to the calculated value presented in Supplementary Fig. 19a, i.e., $\Delta E_{rp,calculated}$ = −22 kcal mol^{-1}.

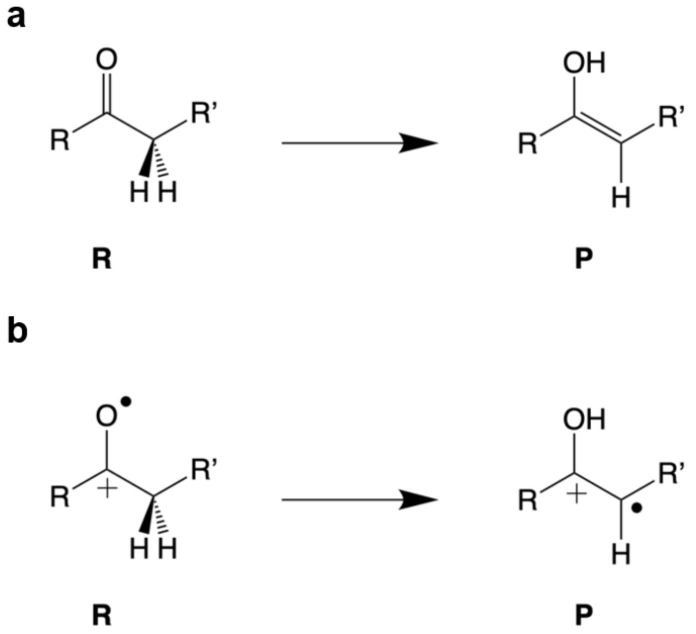

Supplementary Figure 20. Schematic description of different charged states. a,b Schematic description of the tautomerization reaction for (**a**) the neutral, uncharged species and (**b**) the (positively) charged species.

The reason for this discrepancy is that the unpaired (radical) electron in the cation can readily delocalize, and will do so to a much greater extent in the enol-form than in the keto-form, due to the conjugation of the lone pair on the oxygen moiety with the adjacent π-type C–C$^{\bullet+}$ system (cf. Supplementary Fig. 21a). The fingerprints of this resonance delocalization can be seen among others in the bond lengths: both the C–C and C–O bond lengths in the enol are intermediate between a single and double bond (Supplementary Fig. 21b), similar to what one observes for a prototypical delocalized allyl radical. As some of us discussed in detail in a recent contribution, a loss or gain of delocalization is inherently connected to a relative thermodynamic penalty paid throughout the reaction[4]. In the tautomerization reaction under consideration, it is the enol-form that is resonance stabilized compared to the keto-form. Based on the preceding argument, the magnitude of the resonance penalty associated

with the tautomerization reaction can thus be crudely estimated at 17 kcal mol^{-1}, i.e., the magnitude of the discrepancy between the naively estimated and the actual, calculated ΔE_{rp}.

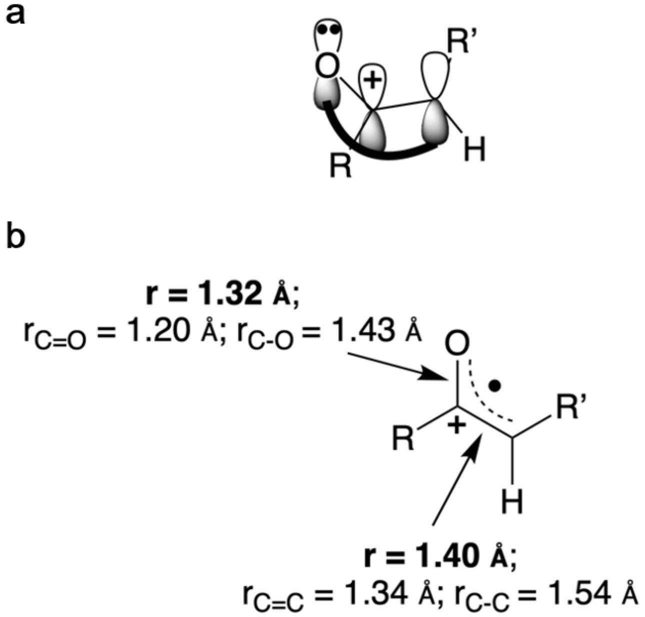

Supplementary Figure 21. Orbital analysis. a The delocalized π-system in the charged enol; **b** the bond lengths for R, R' = Ph.

Next to the switching in thermodynamic preference, the dramatic change in the kinetics of the tautomerization upon oxidation can also be readily understood from a qualitative valence bond (VB) analysis[5]. Central to VB reactivity theory is the construction of so-called valence bond state correlation diagrams (VBSCD), which depict the evolution of individual diabatic states, corresponding to the electronic configuration of the reactant and the product, along the reaction coordinate. In practice, these diabatic states interact and mix as the reaction proceeds, and collectively they give rise to the adiabatic curve, i.e., the full ground-state PES associated with the chemical reaction under consideration (Supplementary Fig. 22).

Within this VB framework, the approximate barrier height associated with a generic chemical reaction can be estimated with the help of the following expression,

$$\Delta E^{\neq} = f_0 G_0 + 0.5\, \Delta E_{rp} - B, \qquad \text{Supplementary Equation (1)}$$

where $f_0 G_0$ corresponds to a fraction (f_0) of the average of the promotion gap on the reactant and product side,

$$G_0 = 0.5\,(G_R + G_P)\ . \qquad \text{Supplementary Equation (2)}$$

Let us now estimate the barrier height associated with the tautomerization reaction for both the uncharged and charged species.

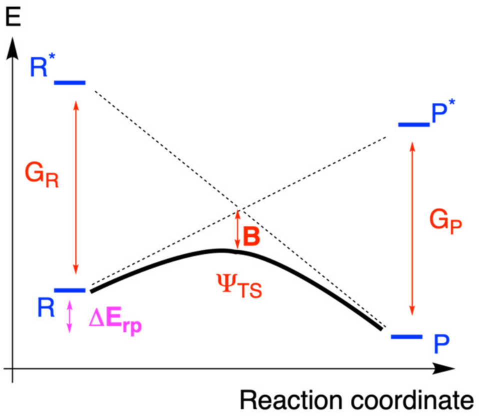

Supplementary Figure 22. VBSCD principle. Generic VBSCDs, depicting the energy curves along the reaction coordinate, which connects the geometry of the reactants (R) to that of the products (P). The reactant diabatic curve connects state R with state P* (the "promoted" product state), and thus depicts the evolution of the (non-interacting) reactant electronic configuration throughout the reaction; the product diabatic curve connects state R* (the "promoted" reactant state) with state P, and thus depicts the evolution of the product configuration. G_R and G_P correspond to the promotion energies separating the diabatic curves, respectively, in the reactant and product geometry. The mixing of these two diabatic VB curves leads to the adiabatic state represented by the bold curve, with B corresponding to the interaction energy between the two curves. ΔE_{rp} corresponds to the thermodynamic driving force, and Ψ_{TS} corresponds to the transition state.

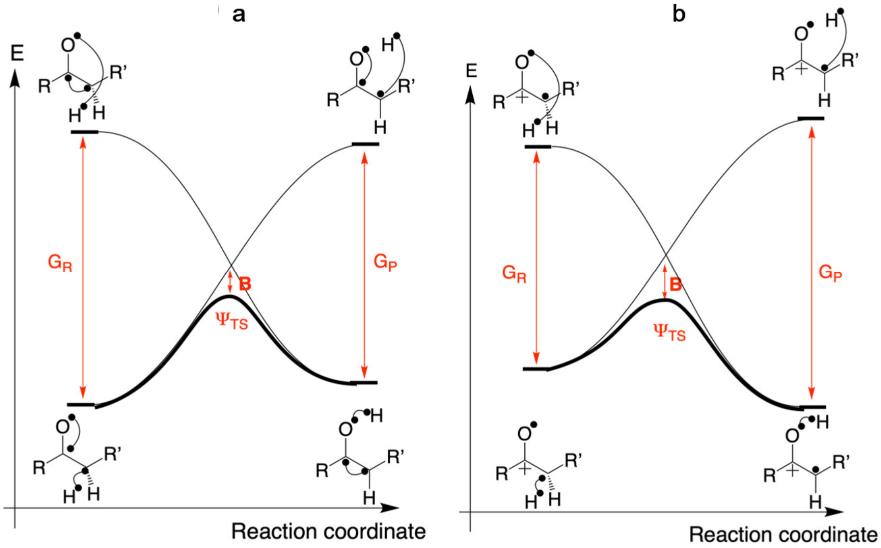

Supplementary Figure 23. VBSCD analysis. a,b Schematic VBSCDs for the tautomerization reaction involving (**a**) the neutral, uncharged species and (**b**), the (positively) charged species.

For the neutral species, a schematic VBSCD with the main representative VB structures for the reactant and product diabatic curves in respectively the reactant and product geometry are shown in Supplementary Fig. 23a.

The (vertical) promotion gap between R and R* can be estimated upon inspection of these two structures. In essence, R can be turned into R* by unpairing the RR'C=O and HRR'C–H bonds and at the same time re-pairing the resulting unpaired electrons residing on the adjacent carbon atoms into an RR'C=CHR" bond (the O and H centers are too far away in the reactant geometry for them to exhibit an actual bonding interaction). The energy required to "(un)pair" the electrons involved in a bond can be expressed as the corresponding singlet-triplet excitation energy. In Supplementary Table 1, these quantities, calculated at B3LYP/def2-TZVP(PCM = tetrachloroethene) level of theory, are presented. For each of the active bonds, approximate ΔE_{ST} values were calculated by taking the geometry of the two bonded atoms without their environment and "passivating" them with H-atoms. For the O–H σ bond, this approach is not feasible, since the O–H bonds in H_2O are much stronger than regular O–H bonds in organic compounds, and passivating the HO· by any other cap than a simple H leads to the formation of a bond that is weaker than the O–H σ bond we want to probe the ΔE_{ST} of. Thus, for this bond, we assumed the ΔE_{ST} to be 4% stronger than the C–H σ bond, in analogy to the difference in BDE between these two bonds.[3]

Supplementary Table 1. E_{ST} values for each of the bonds broken/formed during the promotion from R to R* and from P to P* respectively. The subscripts "R" and "P" denote whether the reactant or product geometry was used in the single-point calculations; the subscripts "broken" and "formed" denote whether the bond is an existing bond that is being broken during the promotion event, or a new bond that is being formed during the promotion.

bond	ΔE_{ST} (kcal mol^{-1})
$[C\text{-}H]_{R,broken}$	241
$[C{=}O]_{R,broken}$	72
$[C{=}C]_{R,formed}$	40
$[O\text{-}H]_{P,broken}$	251
$[C{=}C]_{P,broken}$	95
$[C{=}O]_{P,formed}$	57

Based on the values in Supplementary Table 1, one can estimate G_R to amount to 273 kcal mol^{-1} and G_P to 289 kcal mol^{-1}. Recall that we had already calculated ΔE_{rp} to amount to 8 kcal mol^{-1}. Based on these quantities and taking the common f_0 – value for concerted and radical reactions, 0.3, one ends up with an estimate for the crossing point of the two diabatic curves in the VBSCD of 88 kcal mol^{-1}, i.e., 0.3 × 0.5 × (273 + 289) + 0.5 × 8. Since the actually calculated barrier amounts to 59 – 61 kcal mol^{-1}, depending on the direction of the electric

field, we can estimate the $\boldsymbol{B}$-factor in Supplementary Equation (1) at 27 – 29 kcal mol^{-1}. This (relatively low) value is perfectly in line with what one would expect for a 4-electron, 4-membered ring transition state geometry: due to symmetry reasons, mixing of the individual VB structures is limited in such a situation; taking cyclobutadiene (the most "notorious" square system) for example, $\boldsymbol{B}$ has previously been determined to amount to approximately 21 kcal mol^{-1}.[6]

We can now follow the same approach to estimate the barrier for the charged species (cf. the VBSCD in Supplementary Fig. 19b). With the help of Supplementary Table 1, G_R and G_P can respectively be estimated at 241 and 251 kcal mol^{-1}, since the bond length of the C–H and O-H bond being broken/formed differ by less than 0.004 Å. As discussed above, ΔE_{rp} amounts to −22 kcal mol^{-1}. Setting f_0 again to 0.3, one ends up with an estimate for the crossing point of the two diabatic curves in the VBSCD of 63 kcal mol^{-1}. Our calculations indicate that in reality, $\Delta E^{\neq}$ amounts to 22.9 kcal mol^{-1}. Based on this value, we can estimate that $\boldsymbol{B}$ amounts to approximately 40 kcal mol^{-1}. This is again a reasonable value: it is higher than the typical $\boldsymbol{B}$ values for the square (and thus resonance-hampered) transition states, and it is lower than the typical $\boldsymbol{B}$ values of approximately 50 kcal mol^{-1} previously determined for intramolecular H-abstraction reactions in which the reactants are unrestraint and thus approach optimally, which maximizes the overlap of the involved orbitals, and thus also the magnitude of $\boldsymbol{B}$.[3]

Putting everything together now, we can conclude that dramatic change in the kinetics upon oxidation of the molecular bridge is the collective effect of each of the individual factors in Supplementary Equation (1): the cationic species exhibits lower promotion energy, a bigger thermodynamic driving force and a bigger $\boldsymbol{B}$. The difference in promotion energy results in approximately 12 kcal mol^{-1} barrier lowering, the change in E_{rp} lowers the barrier by an additional 15 kcal mol^{-1}, and $\boldsymbol{B}$ pushes the barrier 15 kcal mol^{-1} down as well.

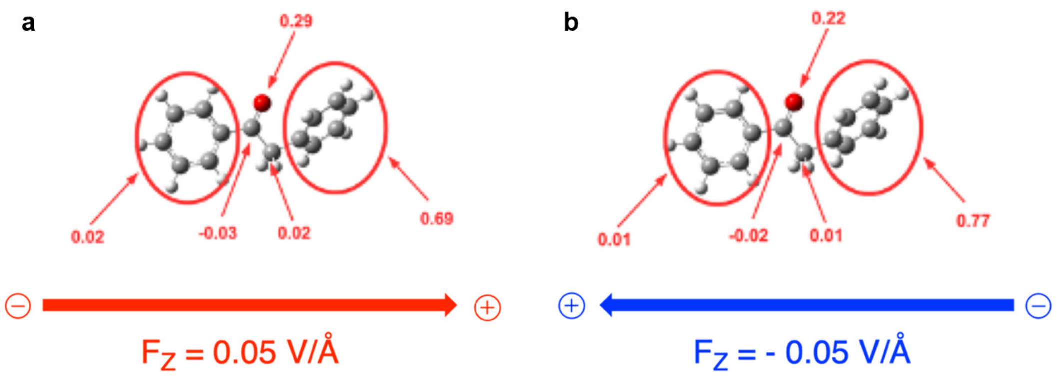

Supplementary Figure 24. Spin density analysis. a,b The atom-condensed Mulliken spin densities, calculated at B3LYP/def2-TZVP (PCM = tetrachloroethene) level-of-theory with F_Z = +0.05 V Å^{-1} (**a**) and F_Z = −0.05 V Å^{-1} (**b**), for the keto-form of the cationic model system.

One final issue that remains to be addressed is the role of the (weak) electric field. As evident from Supplementary Fig. 19a–b, the application of a field of +0.05 V A^{-1} leads to reaction barriers and thermodynamic driving forces that are a couple of kcal mol^{-1} lower (+22.9 and −22.0 kcal mol^{-1}, respectively) than when the field is applied in the opposite direction, i.e., –0.05 V Å^{-1} (+24.9 and −19.4 kcal mol^{-1} respectively). The root cause of these slight shifts is a change in the extent of delocalization in the keto-form: under the influence of a field of +0.05 V Å^{-1}, the spin density on the O-atom (which is the reactive site in the H-abstraction reaction) amounts to 0.29 e, under the influence of a field of −0.05 V Å^{-1}, this spin density decreases to 0.22 e (cf. Supplementary Fig. 24). The higher extent of localization in the positive field means that a bigger gain of delocalization energy is associated with the tautomerization reaction towards the enol under these circumstances. Hence, the thermodynamic driving force increases, and according to the Bell-Evans-Polanyi principle (as well as Supplementary Equation (1)), this will also reduce the reaction barrier.[2]

For the actual molecular bridge with S-anchors included, the same analysis as above is valid. There is however one major complication: upon charging, the Sulfur-moieties carry a significant spin density, i.e., the positive charge is delocalized a lot more than without the anchors. As a result of this charge delocalization, the dramatic effect on thermodynamics and kinetics, observed in Supplementary Fig. 19, is somewhat tempered. Whereas the uncharged PES is not altered meaningfully upon inclusion of the anchors, i.e., the barrier still amounts to approximately 60 kcal mol^{-1} and the keto-form is more stable than the enol-form by approximately 10 kcal mol^{-1}, the charged PES exhibits a significantly smaller thermodynamic driving force towards the enol-form (7 kcal mol^{-1} vs. 22 kcal mol^{-1}) and a significantly higher reaction barrier (35 kcal mol^{-1} vs. 23 kcal mol^{-1}). Supplementary Equation (1) indicates that the bulk of this barrier height increase (7.5 out of 12 kcal mol^{-1}) is caused by the reduced thermodynamic driving force, i.e., the change in the extent of delocalization; the rest most likely stems from a change in the $\boldsymbol{B}$-factor.

Supplementary Note 3. Assessment of the feasibility of the charge transfer event.

To gauge whether a thermal transition from the uncharged to charged Marcus parabola is feasible (cf. Fig. 5b in the main text), and can consequently explain the tautomerization is giving rise to the observed switching behavior in the conductance, we followed the analysis by Nitzan and co-workers.[7] Let us first consider the thermodynamic driving force, i.e., the energy difference between the minimum of the two parabolas in Fig. 5b. This energy difference, ΔE_{MB-MB^+}, can be expressed as follows:

$$\Delta E_{MB^+-MB} = IP(MB) - E_F(electrode) - eV/2 \qquad \text{Supplementary Equation (3)}$$

where IP(MB) corresponds to the ionization potential of the molecular bridge, $E_F(electrode)$ corresponds to the (unbiased) Fermi energy of the electrodes, and V corresponds to the applied voltage.

From the fitted I-V curve, presented in Fig. 3c, this gap can be determined straightforwardly. Our estimation that the Fermi level of the junction is located approximately 0.65 – 0.75 eV away from the HOMO eigenchannel indicates that $IP(MB) - E_F(electrode)$ amounts to 14.9 – 17.3 kcal mol^{-1}. Correspondingly, at a voltage of

0.4 – 0.5 V, $\Delta E_{MB^{+}-MB}$ can be estimated at 9.2 – 12.7 kcal mol^{-1} according to Supplementary Equation (3). Note that these values agree within reasonable bounds with the calculated thermodynamic driving force towards the enol-form on the charged PES (see main text).

Let us now take a look at the potential existence of a thermal barrier separating the uncharged and charged keto-species. According to Marcus theory, the barrier height for a charge transfer event can be estimated to amount to,

$$\Delta E^{\neq}_{MB^{+}-MB} = \frac{(\lambda+\Delta E_{MB^{+}-MB})^2}{4\lambda}, \qquad \text{Supplementary Equation (4)}$$

where λ corresponds to the reorganization energy. In accordance with the methodology used by Nitzan and co-workers,[7] the reorganization energy was estimated from gas-phase calculations on the isolated molecular bridge: the energy difference between E_{MB} and $E_{MB^{+}}$ was determined in the optimal geometry of the uncharged molecular bridge, after which the resulting value was corrected to reflect the actual alignment between the two Marcus parabolas as inferred from the experimental data, i.e., $\Delta E_{MB^{+}-MB}$ = 9.2 – 12.7 kcal mol^{-1} (cf. Supplementary Equation (3)). Using this approach, approximate λ values of 11.3 – 14.8 kcal mol^{-1} could be determined. Plugging these values into Supplementary Equation (4), one ends up with a barrier height in the range of 9.3 – 12.8 kcal mol^{-1}.

As such, the inferred thermodynamic driving force together with the crudely estimated reaction barrier suggests that a transition from the uncharged parabola to the charged parabola can occur at room temperature. Hence, this thermal charge transfer mechanism is a plausible trigger of the tautomerization reaction; the bias window does not need to reach the HOMO peak in the transmission spectrum for the electron transfer to occur.

Supplementary Note 4. Estimation of the alignment of the Fermi level with the HOMO transport channel.

In order to fit the experimental I-V curve, we solve the following equation[8],

$$I(V,T) = \frac{2e}{h}\int_{-\infty}^{\infty} dE\, T(E)\left[f\left(E-E_F-\frac{eV}{2},T\right)-f\left(E-E_F+\frac{eV}{2},T\right)\right], \qquad \text{Supplementary Equation (5)}$$

where

$$f(x,T) = \frac{1}{\exp\left(\frac{x}{k_B T}\right)+1}, \qquad \text{Supplementary Equation (6)}$$

and T(E) is approximated as a single resonance channel, i.e., the HOMO eigenchannel, centered at energy through a Lorentzian function,

$$T(E) = \frac{(\Gamma/2)^2}{(\mathrm{E}-\varepsilon)^2+(\Gamma/2)^2}. \qquad \text{Supplementary Equation (7)}$$

By varying the distance between the resonance and the Fermi level ($E_F - \varepsilon$), as well as the resonance width Γ, one can tune the shape of the calculated I-V curve. An optimal agreement between the calculated and measured I-V curve is reached at $E_F - \varepsilon \approx 0.7\ eV$ and $\Gamma = 0.012\ eV$ (see Fig. 3c in the main text).

Supplementary Note 5. Classification results in different biases.

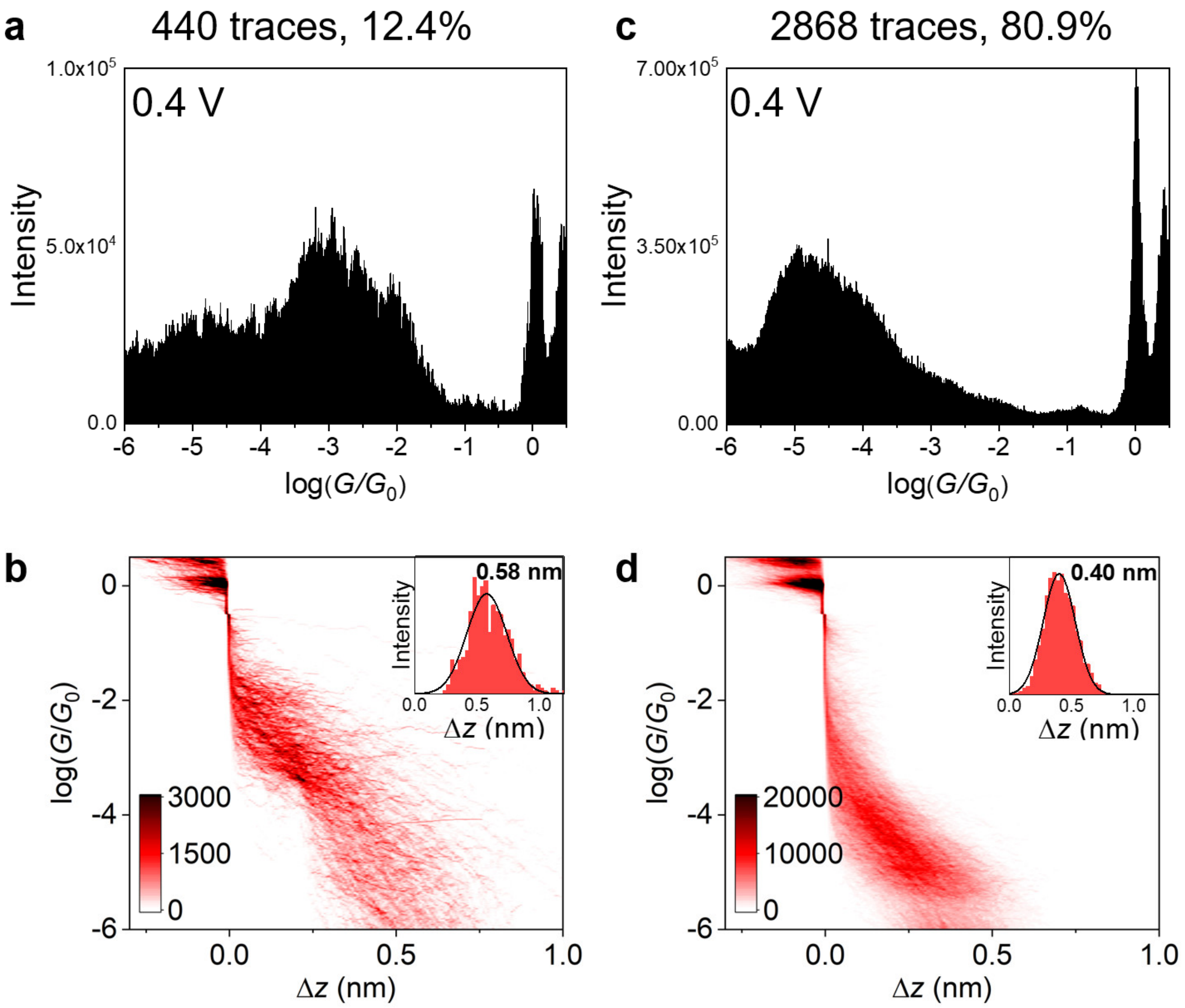

Supplementary Figure 25. The STM-BJ experimental data at 0.4 V bias. a-d The 1D conductance histograms of the high-conductance state (**a**) and low-conductance state (**c**). The 2D conductance histograms of the high-conductance state (**b**) and low-conductance state (**d**) with the insets showing the stretching distance ranging from $10^{-6.0}$ to $10^{-0.3}$ G_0. The color scales represent intensity.

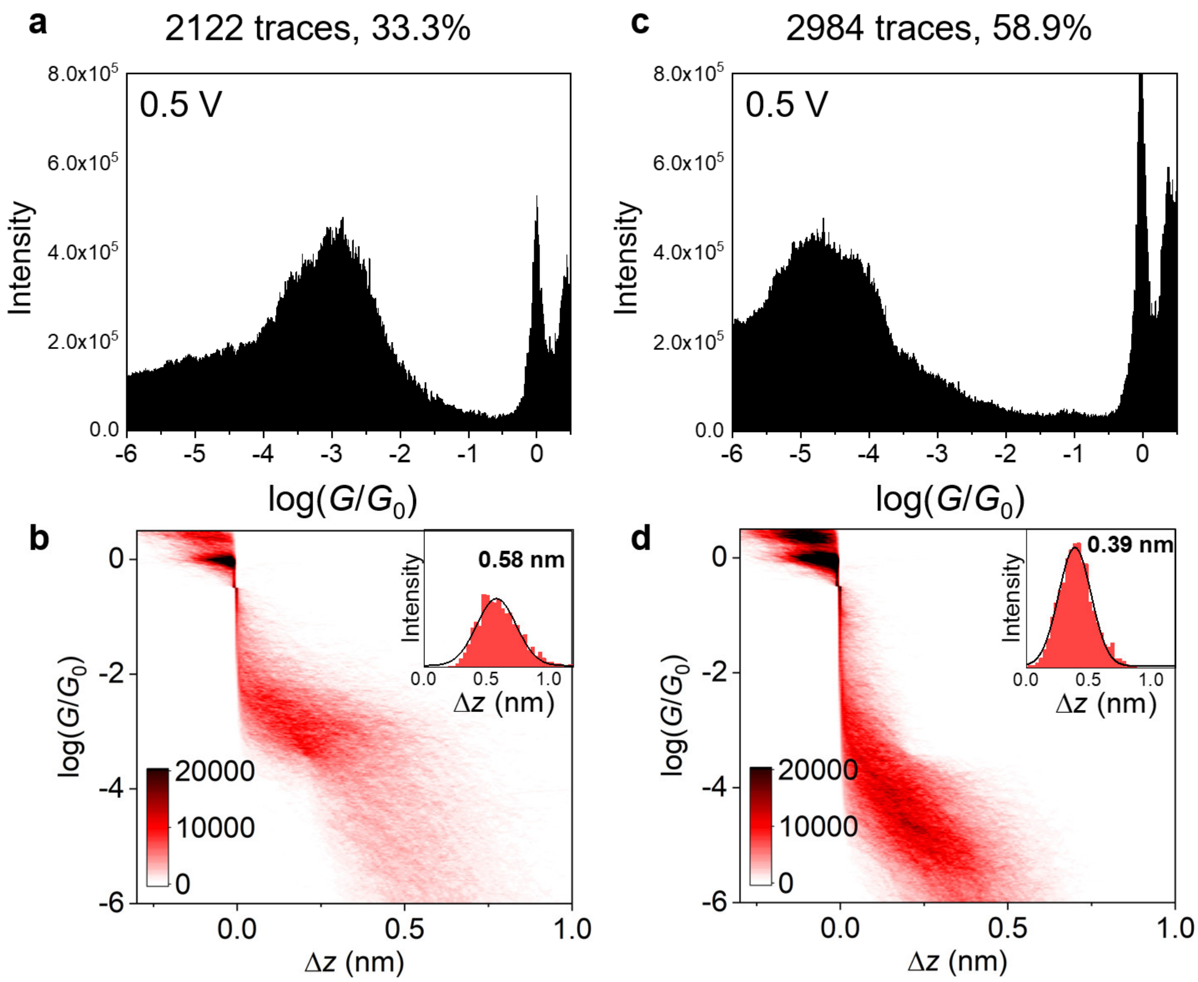

Supplementary Figure 26. The STM-BJ experimental data at 0.5 V bias. a-d The 1D conductance histograms of the high-conductance state (**a**) and low-conductance state (**c**). The 2D conductance histograms of the high-conductance state (**b**) and low-conductance state (**d**) with the insets showing the stretching distance ranging from $10^{-6.0}$ to $10^{-0.3}$ G_0. The color scales represent intensity.

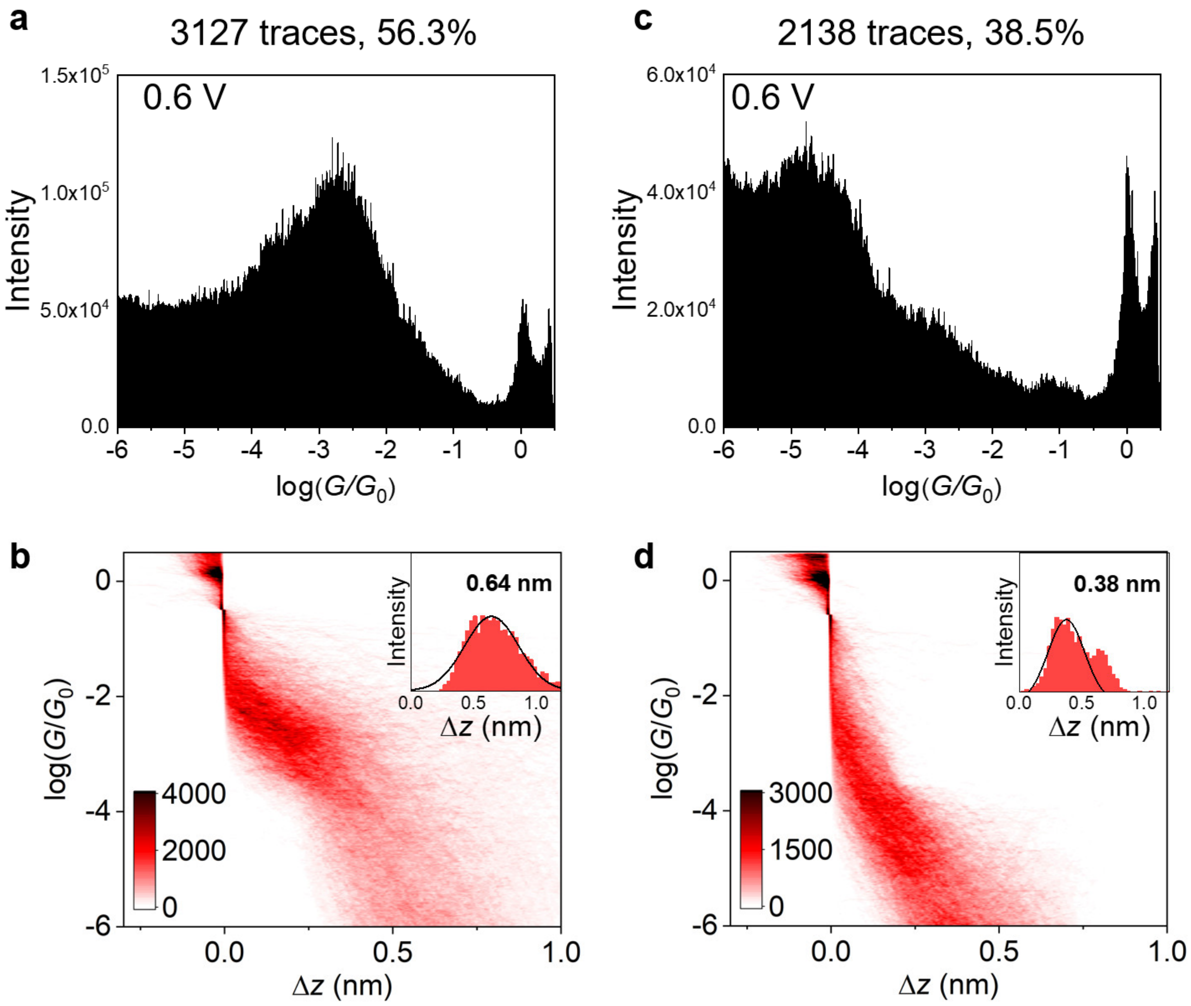

Supplementary Figure 27. The STM-BJ experimental data at 0.6 V bias. a-d The 1D conductance histograms of the high-conductance state (**a**) and low-conductance state (**c**). The 2D conductance histograms of the high-conductance state (**b**) and low-conductance state (**d**) with the insets showing the stretching distance ranging from $10^{-6.0}$ to $10^{-0.3}$ G_0. The color scales represent intensity.

Supplementary Note 6. Covariance analysis.

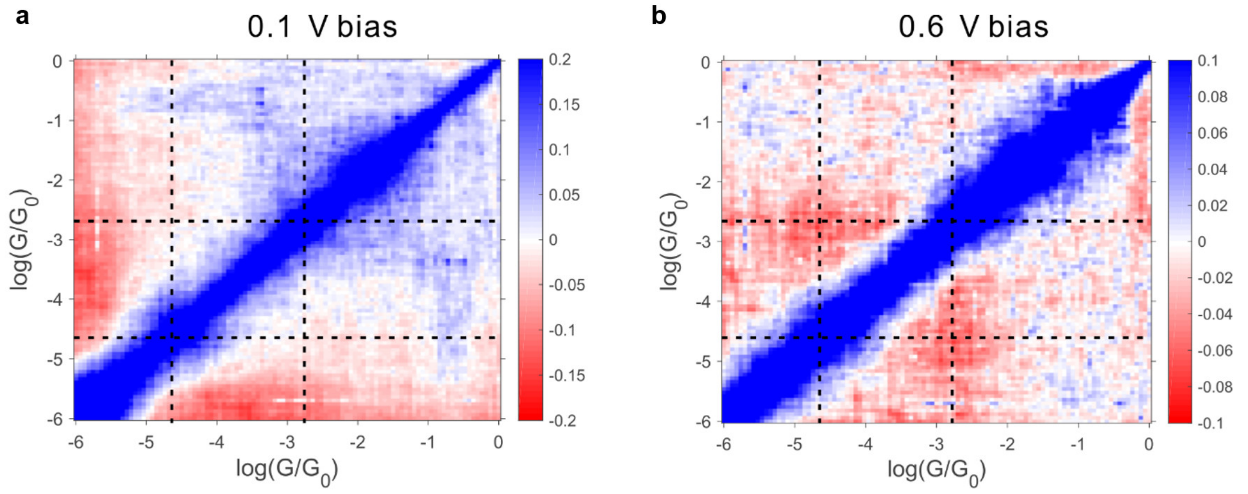

Supplementary Figure 28. Covariance analysis. a,b The 2D covariance histograms of the total data at 0.1 V (**a**) and 0.6 V (**b**) bias. The correlation matrix for the dataset of 0.6 V bias shows a clear negative correlation (red areas) between the high- and low-conductance values, which means that the two conductance plateaus occurred in a mutually exclusive pattern. As a reference, such a negative correlation does not emerge from the dataset at 0.1 V bias.

Supplementary Note 7. Microfabrication of MCBJ chip.

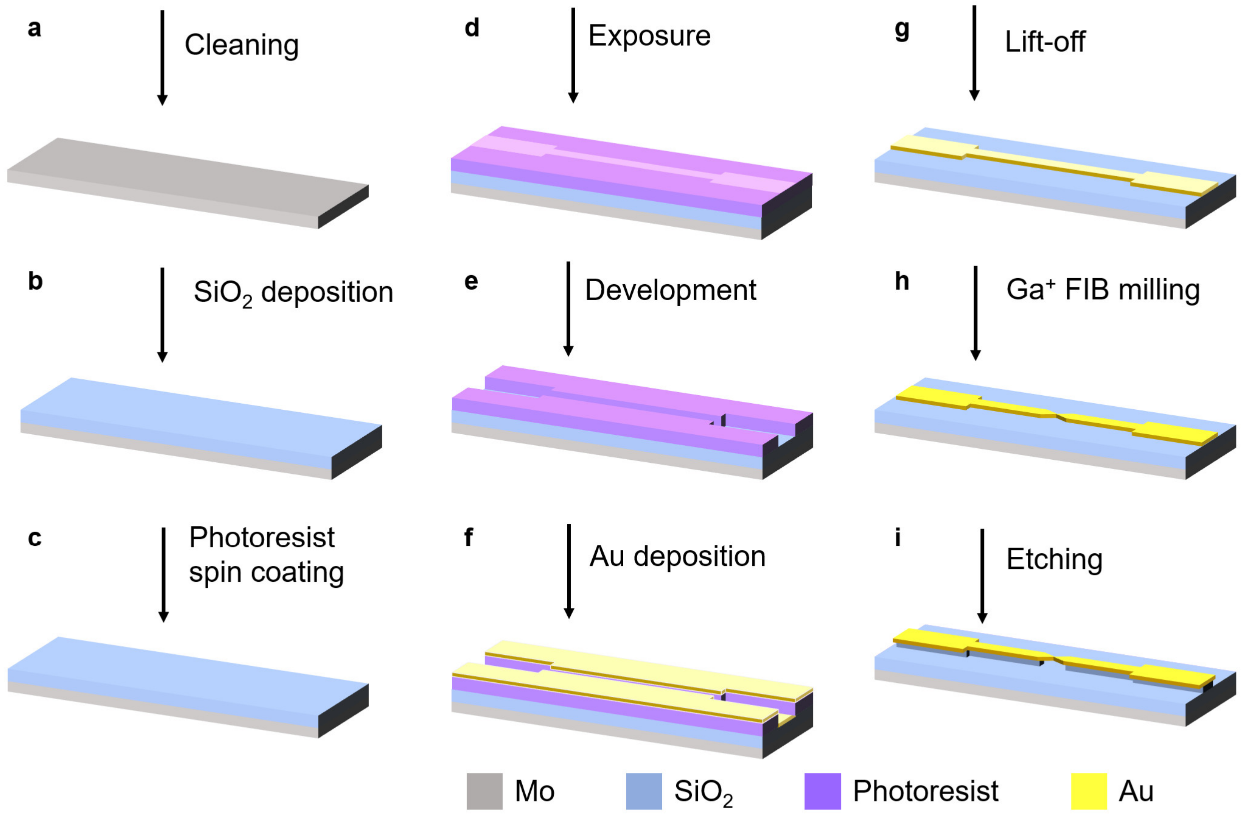

Supplementary Figure 29. The preparation procedure of the microfabrication MCBJ chip. a A polished moly (Mo) substrate (4 inches in diameter and 150 μm in thickness) was cleaned with acetone, ethanol, and deionized water. **b** Then an insulating and sacrificial layer of SiO_2 (2 μm in thickness) was deposited using plasma-enhanced chemical vapor deposition (SI 500D, Sentech Instruments GmbH). **c–e** The electrode patterns with micron linewidth were fabricated by photolithography (MA8BA8, SUSS MicroTec Lithography GmbH). (**e**) An Au film (50 nm in thickness) with Ti (3 nm in thickness) as the adhesion layer serving as electrodes of the MCBJ was fabricated by electron beam evaporation (DE400, DE Technology Inc.) and (**g**) lift-off process. **h** Then we introduced the Ga^+ focus ion beam (FIB) milling (Orion Nanofab, Zeiss) to form narrow electrodes with a width of around 80 nm. **i** Finally, the suspended structures were realized by plasma etching (HAASRODE-E200A, Leuven Instruments) and isotropic wet etching using buffered oxide etchant. The SEM image of the suspended nano bridge is shown in Fig. 4a. The contacting pads were connected to gold wires (0.1 mm in diameter) through the silver conductive paint (SCP03B, Electrolube).

Supplementary Note 8. MCBJ Experiments.

The MCBJ chip was fixed onto a homebuilt MCBJ setup,[9] and the gold wires were connected to the electrical measurement instrument for the electrical characterization. With 0.1 V bias applied at room temperature and atmospheric environment, we first broke the suspended electrode into an adjustable nanogap by moving the pushing pod connected to a motor to bend the substrate of the chip, which can break the gold wire to form a pair of gold electrodes. When there was a sudden decrease in the conductance (from short-circuit current to the noise background of the instrument), we stopped the movement of the pushing pod. Then we restore the connection of the electrodes by slowly lowering the pushing pod to adjust the distance between the pair of gold electrodes. After that, 30 μL solution of compound **1** (1.0 mM) in the solvent mixture of dichloromethane/1,3,5-trimethylbenzene (*v*/*v*,1/9) was added onto the etching position of the MCBJ chip. The solution quickly dried within minutes after adding the solution, and the following MCBJ experiment was performed in the dried sample. The pushing pod was slowly moved up to break the contact of the electrodes until a stable conductance value could be observed (Supplementary Fig. 31) between 10^{-4} and 10^{-5} G_0, which is the representative conductance value of the keto form of compound **1** as we have obtained from the STMBJ experiment. We then stopped the movement of the pushing pod, and performed the I-V characterization on the MCBJ chip.

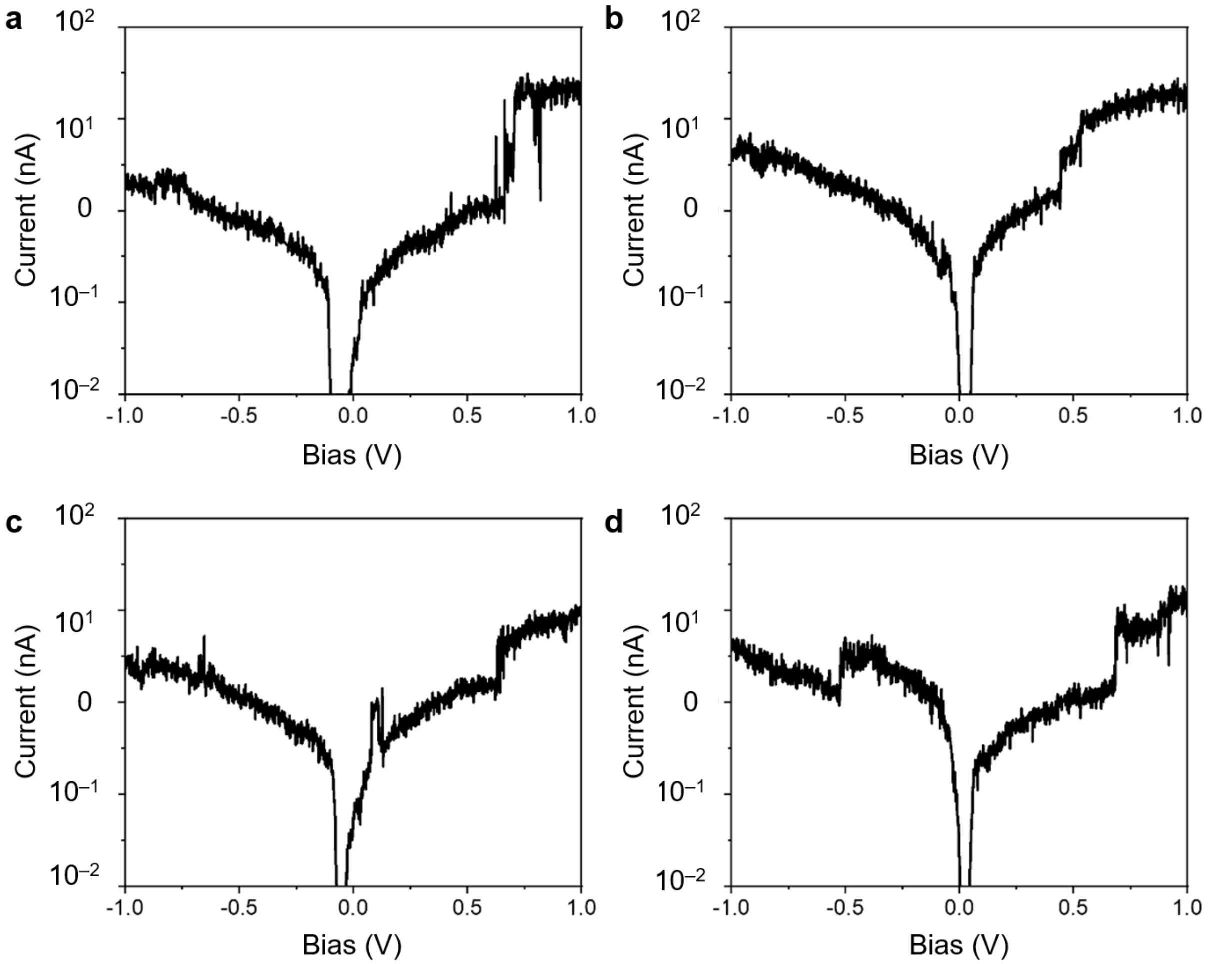

Supplementary Figure 30. MCBJ experiments. a–d I-V Measurements of the single-molecule junction (molecule **1**) formed on the microfabrication MCBJ chip, suggest that this device (also shown in Fig. 4e) can only show keto-enol transformation behavior within the range of the positive bias.

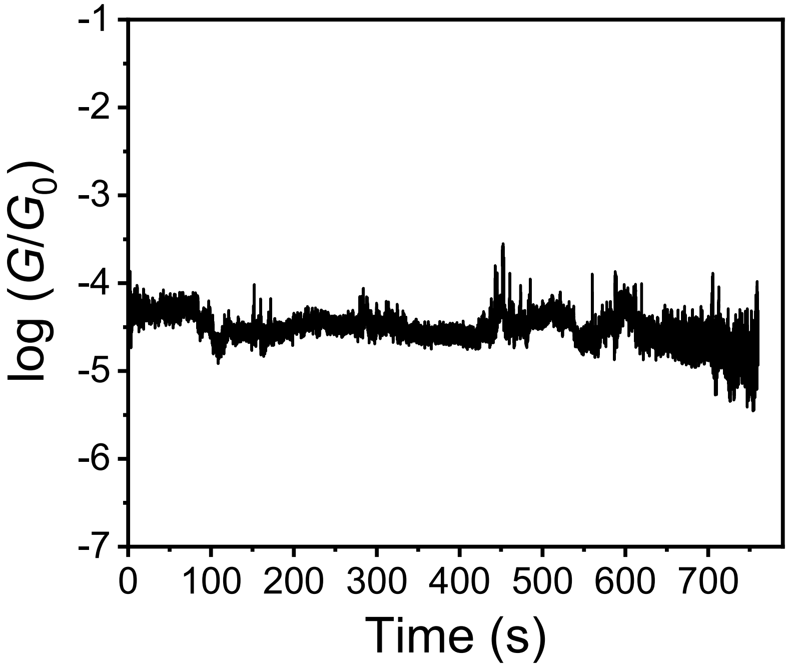

Supplementary Figure 31. MCBJ experiments. The stability of the single-molecule junction (molecule **1** in keto form) with 0.1 V bias was applied in the microfabrication MCBJ chip within a time scale of over 10 minutes.

Supplementary Note 9. Tabulated geometries and energies

Units:

- Coordinates are expressed in Å
- Energies are expressed in atomic units (a.u.)

a) Direct tautomeric switch mechanism (F_z = +0.05 V Å^{-1})

Keto-form of the charged model system without linkers

B3LYP/def2-TZVP geometry:

C	1.210243	0.499384	2.486519
C	1.305255	0.978064	3.799224
C	0.207481	0.917274	4.625983
C	–1.031975	0.435044	4.092236
C	–1.108509	–0.093150	2.788548
H	2.086524	0.511779	1.851273
H	2.254555	1.342480	4.168132
H	–1.947194	0.595554	4.643943
H	–2.050743	–0.473767	2.420193
C	0.270008	1.232148	6.094345
H	–0.303593	2.135629	6.331578
H	1.293230	1.422353	6.418498
C	–0.302210	0.042732	6.845077
O	–0.809042	–0.852607	6.167473
C	–0.256430	–0.041490	8.310263
C	0.310488	0.981524	9.082613
C	–0.798969	–1.169528	8.944399
C	0.331564	0.876250	10.464789
H	0.733762	1.859874	8.615324
C	–0.772007	–1.268823	10.323921
H	–1.236041	–1.955138	8.343915
H	0.767926	1.668335	11.058347
H	–1.189669	–2.140137	10.810407
C	0.000928	–0.046811	1.984752
H	–0.038305	–0.400627	0.963780
C	–0.207520	–0.246694	11.084707
H	–0.188073	–0.326641	12.164004

B3LYP/def2-TZVP energy: –615.8958591

Transition state of the charged model system without linkers

B3LYP/def2-TZVP geometry:

C	0.926946	1.195908	2.625885
C	0.350102	1.778560	3.730473
C	–0.166267	0.972039	4.780871
C	–0.108696	–0.440128	4.652921
C	0.468651	–1.010532	3.542479
H	1.329788	1.812997	1.834594
H	0.292082	2.855995	3.814616
H	–0.516031	–1.070685	5.432414
H	0.518656	–2.086775	3.449788
C	–0.766639	1.615915	5.908116
H	–2.158371	1.690952	5.778388
H	–0.511337	2.655911	6.106273
C	–1.355450	0.910797	7.079997
O	–2.604635	1.053890	6.897485
C	–0.760115	0.245291	8.193374
C	0.641172	0.189587	8.307945
C	–1.575585	–0.345408	9.179017
C	1.215582	–0.445517	9.393794
H	1.265627	0.644646	7.550488
C	–0.990172	–0.978836	10.256384
H	–2.651521	–0.298893	9.080396
H	2.291605	–0.490313	9.489519
H	–1.607732	–1.436861	11.016781
C	0.989028	–0.198669	2.525207
H	1.436209	–0.654696	1.651920
C	0.401375	–1.027892	10.362984
H	0.853907	–1.526078	11.210735

B3LYP/def2-TZVP energy: –615. 8509068

Enol-form of the charged model system without linkers

B3LYP/def2-TZVP geometry:

C	0.855896	1.560319	2.543221
C	0.824901	1.481604	3.914375
C	0.123391	0.429636	4.577436
C	–0.540107	–0.544103	3.774675
C	–0.495848	–0.455270	2.403246
H	1.387672	2.365141	2.054732
H	1.332994	2.227610	4.511719
H	–1.072367	–1.355400	4.242002
H	–0.996908	–1.199775	1.799729
C	0.157716	0.442088	5.989861
H	0.738962	1.237659	6.433295
C	–0.473122	–0.396835	6.918271
C	–0.289434	–0.247252	8.354324
C	0.892706	0.303978	8.882389
C	–1.295382	–0.673374	9.242300
C	1.062956	0.410909	10.250950
H	1.693018	0.610724	8.223981
C	–1.122799	–0.550261	10.608918
H	–2.240274	–1.050050	8.869481
H	1.982817	0.820259	10.645987
H	–1.911430	–0.861896	11.280153
C	0.195566	0.591458	1.779754
H	0.220674	0.648990	0.699456
C	0.057829	–0.012646	11.116899
H	0.192049	0.079491	12.186610
O	–1.296263	–1.330603	6.465032
H	–1.599440	–1.921234	7.171103

B3LYP/def2-TZVP energy: –615.9287424

Keto-form of the uncharged model system without linkers

B3LYP/def2-TZVP geometry:

C	1.227624	0.171861	2.513355
C	1.248212	0.378836	3.887913
C	0.084818	0.720972	4.576363
C	–1.101877	0.853891	3.855998
C	–1.127663	0.648006	2.481556
H	2.141028	–0.091476	1.994836
H	2.180459	0.273668	4.431176
H	–2.015614	1.121734	4.374218
H	–2.057810	0.757081	1.938019
C	0.107772	0.938481	6.065345
H	–0.626303	1.697974	6.350247
H	1.079587	1.331938	6.377999
C	–0.172593	–0.313729	6.897989
O	–0.386211	–1.388294	6.378309
C	–0.175083	–0.178556	8.393847
C	0.058268	1.034810	9.047593
C	–0.423116	–1.322415	9.160170
C	0.043414	1.102155	10.435749
H	0.252682	1.935439	8.482120
C	–0.437520	–1.255530	10.544554
H	–0.602833	–2.257091	8.646659
H	0.225189	2.047982	10.930128
H	–0.630377	–2.148300	11.126019
C	0.038326	0.305622	1.805565
H	0.020853	0.147687	0.734732
C	–0.204176	–0.041535	11.186106
H	–0.215371	0.011804	12.267675

B3LYP/def2-TZVP energy: –616.17687

Transition state of the uncharged model system without linkers

B3LYP/def2-TZVP geometry:

C	1.026955	1.298271	2.623581
C	0.569364	1.730782	3.861351
C	–0.187157	0.892246	4.693860
C	–0.462648	–0.403212	4.229984
C	–0.000353	–0.832827	2.993157
H	1.607297	1.972992	2.005834
H	0.800072	2.736281	4.193803
H	–1.050264	–1.082760	4.834312
H	–0.227066	–1.840047	2.664718
C	–0.651492	1.431662	6.001945

H	–2.125497	1.157209	5.837039
H	–0.048888	2.266890	6.348281
C	–1.205254	0.568994	7.007496
O	–2.400516	0.227086	6.649573
C	–0.651790	0.134111	8.280208
C	0.704854	0.338118	8.565345
C	–1.465859	–0.515468	9.220015
C	1.237253	–0.098868	9.768651
H	1.334651	0.823821	7.832338
C	–0.925899	–0.962758	10.414463
H	–2.514783	–0.656482	8.998217
H	2.285880	0.058368	9.984599
H	–1.553882	–1.469013	11.136096
C	0.746343	0.011644	2.177306
H	1.101110	–0.326789	1.212577
C	0.423715	–0.751678	10.690507
H	0.842033	–1.097363	11.627588

B3LYP/def2-TZVP energy: –616.0739266

Enol-form of the uncharged model system without linkers

B3LYP/def2-TZVP geometry:

C	0.816654	1.564365	2.507539
C	0.784849	1.485942	3.891425
C	0.109999	0.445774	4.555346
C	–0.539443	–0.517046	3.762153
C	–0.502997	–0.436294	2.376621
H	1.345039	2.378526	2.027122
H	1.291786	2.242365	4.479534
H	–1.071989	–1.326944	4.234722
H	–1.009787	–1.191131	1.787820
C	0.143407	0.443368	6.013879
H	0.691635	1.267428	6.451835
C	–0.416011	–0.400184	6.903129
C	–0.258518	–0.253158	8.364804
C	0.940566	0.202069	8.928743
C	–1.313069	–0.582510	9.227934
C	1.073136	0.338661	10.302888
H	1.779417	0.426709	8.283657
C	–1.176547	–0.448329	10.604180
H	–2.261362	–0.911145	8.820424
H	2.011374	0.686652	10.716737
H	–2.009075	–0.696825	11.250551
C	0.171694	0.600340	1.739421
H	0.192932	0.656953	0.658613
C	0.015879	0.013843	11.148343
H	0.123237	0.116358	12.220734
O	–1.225969	–1.424664	6.485469
H	–1.321058	–2.069956	7.196275

B3LYP/def2-TZVP energy: –616.1642926

Keto-form of the charged model system with linkers

B3LYP/def2-TZVP geometry:

16	0.080020	0.116116	0.157019
16	–0.102691	–0.057299	12.908037
C	1.365005	0.475950	2.590557
C	1.295282	0.691730	3.944452
C	0.056668	0.822461	4.605482
C	–1.128579	0.722957	3.852033
C	–1.085031	0.511659	2.498910
C	0.169777	0.381937	1.840280
H	2.327419	0.382173	2.110646
H	2.210907	0.763293	4.516871
H	–2.085745	0.813387	4.347145
H	–1.999508	0.439163	1.924582
C	0.004914	1.040521	6.078386
H	–0.734767	1.811901	6.316491
H	0.967821	1.392275	6.448687
C	–0.407414	–0.228760	6.871212
O	–0.788519	–1.218810	6.283773
C	–0.322355	–0.166994	8.348250
C	0.023713	0.993317	9.055215
C	–0.607836	–1.327275	9.077844
C	0.076886	0.992780	10.435002
H	0.248885	1.914296	8.534780
C	–0.552942	–1.337873	10.459338
H	–0.876851	–2.226103	8.539925
C	–0.208863	–0.174557	11.159779

H	0.342295	1.900484	10.962196
H	–0.779781	–2.253085	10.986056
C	1.771931	–0.033001	–0.441194
H	2.323377	0.887949	–0.260763
H	2.263411	–0.886167	0.023000
H	1.678887	–0.200903	–1.512180
C	–0.525497	–1.714612	13.498425
H	–1.538810	–1.991972	13.213223
H	0.188580	–2.457151	13.146691
H	–0.467166	–1.653216	14.583913

B3LYP/def2-TZVP energy: –1491.0345859

Transition state of the charged model system with linkers

B3LYP/def2-TZVP geometry:

C	1.031295	1.145164	2.684134
C	0.458938	1.722212	3.781106
C	–0.140007	0.932564	4.801696
C	–0.136594	–0.476963	4.629961
C	0.435182	–1.061670	3.529379
H	1.487353	1.764404	1.922315
H	0.460797	2.799774	3.882126
H	–0.596070	–1.109198	5.378816
H	0.420993	–2.137343	3.436556
C	–0.777391	1.575421	5.904246
H	–2.213106	1.599884	5.799883
H	–0.503334	2.607225	6.113856
C	–1.386988	0.863331	7.062390
O	–2.645588	0.972251	6.871292
C	–0.797106	0.224123	8.187080
C	0.604017	0.208747	8.356144
C	–1.607015	–0.387239	9.165837
C	1.167146	–0.386834	9.457648
H	1.240839	0.676009	7.616746
C	–1.044866	–0.987841	10.268049
H	–2.681559	–0.381321	9.042160
H	2.242206	–0.389298	9.581502
H	–1.688088	–1.451020	11.000715
C	1.033667	–0.265177	2.528452
C	0.352832	–0.995204	10.435010
16	1.179727	–1.711162	11.786751
16	1.780073	–0.864796	1.098263
C	–0.132760	–2.382839	12.836064
H	–0.703503	–3.149332	12.315544
H	0.386630	–2.837520	13.677842
H	–0.787441	–1.595692	13.205304
C	1.599086	–2.661201	1.168528
H	0.549029	–2.946852	1.174453
H	2.064787	–3.026403	0.255243
H	2.123498	–3.073346	2.028353

B3LYP/def2-TZVP energy: –1490.9681977

Enol-form of the charged model system with linkers

B3LYP/def2-TZVP geometry:

16	–2.051937	1.707286	0.439839
16	1.077545	–0.413513	12.692711
C	–0.454101	0.569098	2.414785
C	–0.179418	0.307821	3.734047
C	–1.018495	0.785995	4.778984
C	–2.129667	1.589787	4.393985
C	–2.416083	1.841151	3.082197
C	–1.588671	1.325800	2.053997
H	0.222427	0.203634	1.656820
H	0.742505	–0.210152	3.961985
H	–2.772819	1.992884	5.165713
H	–3.278690	2.440539	2.820493
C	–0.807733	0.563696	6.164376
H	–1.375634	1.201211	6.826444
C	–0.029459	–0.402441	6.792083
C	0.234553	–0.415387	8.212200
C	–0.011157	0.707596	9.032642
C	0.775891	–1.566219	8.817600
C	0.257051	0.672780	10.378498
H	–0.390688	1.625458	8.606667
C	1.043560	–1.606792	10.169377
H	0.973957	–2.439178	8.213251
C	0.787526	–0.488129	10.977080
H	0.067771	1.548828	10.985357

H	1.448684	–2.512778	10.594516
C	–0.809413	0.935455	–0.620916
H	0.175828	1.361441	–0.441755
H	–0.801072	–0.144744	–0.490301
H	–1.122898	1.171238	–1.636090
C	1.756175	–2.035289	13.118338
H	1.037571	–2.828701	12.921304
H	2.691333	–2.221752	12.593814
H	1.951175	–1.988749	14.188241
O	0.515817	–1.432465	6.127623
H	0.123880	–1.533263	5.249273

B3LYP/def2-TZVP energy: –1491.044299

Keto-form of the uncharged model system with linkers

B3LYP/def2-TZVP geometry:

16	–0.027061	0.248391	0.042299
16	–0.343687	0.009927	12.947168
C	1.298770	0.411040	2.513370
C	1.290870	0.581915	3.894470
C	0.108180	0.790602	4.596242
C	–1.083012	0.824506	3.868011
C	–1.091292	0.655691	2.494157
C	0.103855	0.445498	1.794958
H	2.242604	0.254587	2.011138
H	2.233816	0.551725	4.428969
H	–2.023237	0.985788	4.382897
H	–2.030356	0.687895	1.954974
C	0.106726	0.967949	6.089458
H	–0.651600	1.699645	6.384628
H	1.063052	1.380478	6.424726
C	–0.148799	–0.313554	6.886985
O	–0.312449	–1.382464	6.334631
C	–0.190760	–0.214438	8.378511
C	–0.014455	0.986106	9.077332
C	–0.419220	–1.377550	9.117942
C	–0.066026	1.019901	10.459511
H	0.164868	1.910996	8.546827
C	–0.472645	–1.355507	10.501713
H	–0.556174	–2.308207	8.583836

C	–0.296642	–0.151799	11.191754
H	0.072205	1.960260	10.978857
H	–0.651737	–2.278179	11.034267
C	1.678767	–0.010881	–0.500277
H	2.304811	0.853345	–0.282199
H	2.106135	–0.910200	–0.058886
H	1.620809	–0.140587	–1.579900
C	–0.660983	–1.670040	13.539465
H	–1.619221	–2.044840	13.183036
H	0.140788	–2.351039	13.258164
H	–0.693474	–1.590393	14.625031

B3LYP/def2-TZVP energy: –1491.2729931

Transition state of the uncharged model system with linkers

B3LYP/def2-TZVP geometry:

16	–0.264784	1.322037	0.507534
16	–1.699329	0.980629	12.051796
C	1.410895	1.332208	2.638965
C	1.862074	0.984662	3.900396
C	1.156772	0.086558	4.717202
C	–0.029455	–0.441988	4.194824
C	–0.489835	–0.093694	2.929999
C	0.222764	0.798132	2.128845
H	1.988843	2.027156	2.040985
H	2.786343	1.418587	4.264143
H	–0.611950	–1.146914	4.775051
H	–1.416051	–0.528771	2.581695
C	1.715385	–0.248034	6.055591
H	2.376599	0.525002	6.438544
H	1.786783	–1.747385	5.904200
C	0.938751	–0.975466	7.023075
O	0.894762	–2.221003	6.663252
C	0.323785	–0.530865	8.253965
C	0.206681	0.838264	8.545888
C	–0.195102	–1.458656	9.168080
C	–0.407019	1.260935	9.705894
H	0.587835	1.566827	7.842914
C	–0.823856	–1.040839	10.326382

H	–0.095037	–2.514721	8.957049
C	–0.935606	0.326362	10.611676
H	–0.494300	2.319752	9.914780
H	–1.216323	–1.781340	11.007417
C	–2.307149	–0.473937	12.939605
H	–3.035642	–1.025045	12.347250
H	–1.490735	–1.126700	13.244135
H	–2.797468	–0.081250	13.828981
C	–1.742723	0.340000	0.156366
H	–2.555857	0.574253	0.842454
H	–2.042349	0.619223	–0.852647
H	–1.526344	–0.727414	0.178284

B3LYP/def2-TZVP energy: –1491.170376

Enol-form of the uncharged model system with linkers

B3LYP/def2-TZVP geometry:

16	–2.219567	1.472522	0.373501
16	1.083477	–0.493651	12.756099
C	–0.440036	0.683974	2.398052
C	–0.108415	0.514783	3.738018
C	–0.996308	0.857452	4.769080
C	–2.229310	1.413362	4.380859
C	–2.566030	1.584863	3.053078
C	–1.677679	1.213168	2.033355
H	0.287414	0.411815	1.646853
H	0.888618	0.161056	3.971229
H	–2.937553	1.709318	5.145799
H	–3.528157	2.011569	2.796005
C	–0.702764	0.704116	6.192995
H	–1.217567	1.390226	6.851943
C	0.058314	–0.241230	6.784863
C	0.319814	–0.313908	8.231055
C	0.184766	0.803351	9.069163
C	0.725038	–1.515952	8.818378
C	0.417791	0.713363	10.427867
H	–0.083218	1.763933	8.650142
C	0.962612	–1.613753	10.183945
H	0.849352	–2.391456	8.197345
C	0.809004	–0.500611	11.008970
H	0.311842	1.596306	11.046648
H	1.267795	–2.567294	10.590205
C	–0.837811	0.892145	–0.639496
H	0.062239	1.479538	–0.463623
H	–0.639324	–0.166185	–0.476149
H	–1.157127	1.036300	–1.670445
C	1.626575	–2.180028	13.122319
H	0.851973	–2.910184	12.891794
H	2.546342	–2.426689	12.593646
H	1.819144	–2.195537	14.194110
O	0.639743	–1.273850	6.101017
H	0.274326	–1.310685	5.206558

B3LYP/def2-TZVP energy: –1491.2619616

b) Alternative H_2O-assisted mechanism

Keto-form without linkers (F_z = –0.05 V Å^{-1})

B3LYP/def2-TZVP geometry:

C	2.610119	–1.936590	1.319557
C	1.718024	–0.911861	1.616759
C	1.455301	0.098861	0.691928
C	2.108714	0.061239	–0.541844
C	2.997927	–0.963987	–0.842165
C	3.251538	–1.967566	0.086872
H	2.804427	–2.708488	2.053661
H	1.229562	–0.893566	2.584072
H	1.925490	0.840421	–1.271541
H	3.496413	–0.975625	–1.803542
C	0.510103	1.237457	1.040516
H	1.082428	2.158898	1.167665
H	0.020125	1.025348	1.990557
C	–0.527683	1.490875	–0.039640
O	–0.354614	2.386167	–0.857867
C	–1.753177	0.646655	–0.117320
C	–1.979849	–0.429124	0.747038
C	–2.713670	0.948145	–1.091323
C	–3.138510	–1.187055	0.638967
H	–1.251805	–0.687978	1.501573
C	–3.871825	0.195598	–1.194235

H	–2.533582	1.781728	–1.755893
C	–4.086394	–0.874987	–0.328789
H	–3.301896	–2.019051	1.311470
H	–4.610877	0.441054	–1.946244
O	1.663730	4.323341	–1.026284
H	0.947491	3.657319	–0.983013
H	1.223056	5.178038	–1.080762
O	2.873231	3.917532	1.492939
H	3.706756	3.453754	1.364257
H	2.542157	4.114331	0.595057
H	–4.991958	–1.463175	–0.408457
H	3.945889	–2.764758	–0.146443

B3LYP/def2-TZVP energy = –769.1291904355553

TS without linkers (F_z = –0.05 V Å^{-1})

B3LYP/def2-TZVP geometry:

C	3.930923	–1.357027	0.937541
C	2.567949	–1.175678	1.117796
C	1.793063	–0.435040	0.206894
C	2.460179	0.114997	–0.902047
C	3.826023	–0.073582	–1.082957
C	4.574490	–0.803056	–0.165707
H	4.493650	–1.933611	1.661762
H	2.083095	–1.617835	1.980982
H	1.893773	0.667962	–1.637385
H	4.308162	0.351789	–1.955638
C	0.346518	–0.303383	0.460306
H	–0.059394	–1.096166	1.077114
C	–0.582398	0.388732	–0.343738
O	–0.288256	1.365009	–1.108062
C	–2.043111	0.050122	–0.229569
C	–2.504704	–1.212417	0.157136
C	–2.984866	1.033025	–0.552062
C	–3.866517	–1.477748	0.235415
H	–1.799973	–2.003383	0.375436
C	–4.345392	0.772046	–0.466272
H	–2.628335	2.002384	–0.872537
C	–4.791692	–0.485230	–0.069922

H	–4.205302	–2.463194	0.529909
H	–5.059796	1.548927	–0.709494
O	1.087291	3.130363	0.070293
H	0.609090	2.443948	–0.536956
H	2.020776	3.137424	–0.174824
H	0.887750	2.527031	1.257790
O	0.654326	1.836353	2.093084
H	0.417195	0.908486	1.540636
H	–0.124883	2.132591	2.584821
H	5.638695	–0.941620	–0.308128
H	–5.852680	–0.691932	–0.005329

B3LYP/def2-TZVP energy = –769.0814798359439

Enol-form without linkers (F_z = –0.05 V Å^{-1})

B3LYP/def2-TZVP geometry:

C	4.072798	–1.327019	0.207968
C	2.687003	–1.351556	0.278419
C	1.908801	–0.290885	–0.215210
C	2.586705	0.785547	–0.814145
C	3.974207	0.807731	–0.885795
C	4.727354	–0.243219	–0.369344
H	4.643933	–2.157190	0.604964
H	2.188184	–2.202822	0.726430
H	2.022007	1.600837	–1.246283
H	4.469147	1.647054	–1.359328
C	0.451686	–0.388817	–0.136060
H	1.859895	2.736245	2.517688
H	0.065774	–1.398518	–0.082961
C	–0.469999	0.598427	–0.069862
O	–0.194178	1.922204	0.054769
C	–1.924043	0.325419	–0.092962
C	–2.452387	–0.799066	–0.742686
C	–2.811745	1.201760	0.542409
C	–3.817195	–1.056898	–0.721980
H	–1.793077	–1.462015	–1.287195
C	–4.176783	0.943792	0.559064
H	–2.419851	2.083131	1.029826
C	–4.686180	–0.188604	–0.066231

H	–4.204488	–1.930134	–1.232036
H	–4.844494	1.629536	1.065571
O	2.002780	2.723819	1.563976
H	0.656761	2.090952	0.512707
H	2.777722	2.166502	1.416135
O	–1.825277	–3.274702	1.500964
H	–2.288123	–2.527050	1.101548
H	–1.668627	–3.011019	2.414394
H	–5.750041	–0.388452	–0.053465
H	5.808183	–0.222021	–0.425571

B3LYP/def2-TZVP energy =
–769.1112264357016

Keto-form without linkers (F_z = +0.05 V Å^{-1})

B3LYP/def2-TZVP geometry:

C	2.598709	–1.972845	1.274210
C	1.727429	–0.934252	1.584958
C	1.464959	0.079661	0.663552
C	2.097594	0.031241	–0.580623
C	2.965476	–1.007966	–0.894667
C	3.218737	–2.014740	0.030972
H	2.791464	–2.749133	2.004396
H	1.252030	–0.908732	2.558845
H	1.914745	0.811843	–1.308741
H	3.445170	–1.029599	–1.865062
C	0.537733	1.228911	1.023896
H	1.115947	2.147658	1.141582
H	0.059697	1.023802	1.981795
C	–0.515591	1.491924	–0.041060
O	–0.350055	2.393160	–0.852818
C	–1.742197	0.649252	–0.108468
C	–1.972796	–0.412659	0.772121
C	–2.698480	0.935494	–1.091177
C	–3.131842	–1.170690	0.672144
H	–1.246228	–0.661518	1.531600
C	–3.856255	0.181170	–1.187489
H	–2.514425	1.755582	–1.771160
C	–4.075177	–0.874509	–0.305324
H	–3.297368	–1.993998	1.355007
H	–4.588898	0.411142	–1.950275
O	1.745120	4.247799	–0.947184
H	0.993341	3.621471	–0.955655
H	1.432102	5.050154	–1.377370
O	2.728444	4.147846	1.674852
H	3.575076	3.701349	1.571601
H	2.406596	4.284235	0.761221
H	3.894296	–2.824036	–0.215220
H	–4.978712	–1.466066	–0.382939

B3LYP/def2-TZVP energy =
–769.1296896355511

TS without linkers (F_z = +0.05 V Å^{-1})

B3LYP/def2-TZVP geometry:

C	3.925583	–1.331541	0.938059
C	2.568589	–1.132814	1.146410
C	1.778569	–0.411989	0.233678
C	2.422684	0.100867	–0.905544
C	3.782138	–0.103634	–1.114231
C	4.546602	–0.813894	–0.194977
H	4.500807	–1.896469	1.661972
H	2.099659	–1.549034	2.031284
H	1.844660	0.634936	–1.646229
H	4.244761	0.289328	–2.011878
C	0.335747	–0.265072	0.513349
H	–0.070111	–1.068414	1.116865
C	–0.597659	0.427426	–0.289967
O	–0.308873	1.421272	–1.031009
C	–2.053146	0.066918	–0.203272
C	–2.497877	–1.213993	0.139691
C	–3.005593	1.041676	–0.519260
C	–3.855723	–1.506654	0.179416
H	–1.781973	–1.998345	0.345351
C	–4.362176	0.753093	–0.471066
H	–2.661303	2.024274	–0.811393
C	–4.792415	–0.523219	–0.120142
H	–4.182091	–2.508028	0.431163
H	–5.085620	1.520819	–0.716053
O	1.179164	3.097670	0.091355

H	0.643543	2.426865	–0.502730
H	2.106480	3.047410	–0.172790
H	0.969077	2.537046	1.252381
O	0.686919	1.868048	2.124583
H	0.433527	0.928435	1.571228
H	–0.112811	2.199596	2.556029
H	5.604764	–0.969129	–0.362440
H	–5.850252	–0.753165	–0.093548

B3LYP/def2-TZVP energy =
–769.0788674359652

Enol-form without linkers (F_z = +0.05 V Å^{-1})

B3LYP/def2-TZVP geometry:

C	3.851911	–1.100139	1.436619
C	2.464969	–1.058946	1.467738
C	1.727294	–0.435526	0.448140
C	2.446831	0.119811	–0.624792
C	3.835305	0.076458	–0.655713
C	4.547825	–0.526421	0.377335
H	4.391562	–1.587785	2.239216
H	1.933971	–1.517277	2.293774
H	1.914976	0.566662	–1.454439
H	4.362068	0.500218	–1.501910
C	0.264135	–0.453250	0.518700
H	1.818253	3.490287	1.165201
H	–0.157924	–1.265719	1.096057
C	–0.625527	0.442527	0.024543
O	–0.295959	1.618335	–0.560775
C	–2.090282	0.240977	0.097866
C	–2.658733	–1.037833	0.163900
C	–2.945034	1.350362	0.091885
C	–4.034614	–1.197910	0.249338
H	–2.026852	–1.914232	0.111749
C	–4.321353	1.186875	0.177039
H	–2.519639	2.341226	0.021440
C	–4.872744	–0.086940	0.260217
H	–4.454577	–2.195178	0.289151
H	–4.965220	2.057607	0.173516
O	1.932373	3.042564	0.319033
H	0.566917	1.970756	–0.255292
H	2.703716	2.469473	0.420642
O	–0.169748	–2.965902	–1.696091
H	–0.686058	–2.732203	–2.475475
H	0.050773	–2.120607	–1.281671
H	5.629140	–0.563506	0.346532
H	–5.946394	–0.214618	0.317502

B3LYP/def2-TZVP energy =
–769.1095979357149

Keto-form with linkers (F_z = –0.05 V Å^{-1})

B3LYP/def2-TZVP geometry:

C	2.552961	–2.001188	1.118963
C	1.690895	–0.970654	1.482102
C	1.426540	0.095084	0.627240
C	2.058661	0.102278	–0.620054
C	2.916106	–0.918624	–0.994006
C	3.175537	–1.988297	–0.128261
H	2.729243	–2.803965	1.820166
H	1.226657	–1.001585	2.461101
H	1.883624	0.919937	–1.308557
H	3.394889	–0.885237	–1.965349
C	0.517574	1.233559	1.055046
H	1.110559	2.137800	1.208192
H	0.050398	0.985840	2.008226
C	–0.544549	1.556918	0.016117
O	–0.374589	2.493472	–0.759665
C	–1.773563	0.734784	–0.077170
C	–2.021508	–0.367509	0.752588
C	–2.737936	1.067130	–1.035332
C	–3.181362	–1.106909	0.625608
H	–1.301723	–0.662112	1.502129
C	–3.904612	0.336227	–1.167727
H	–2.556402	1.917179	–1.678733
C	–4.141916	–0.766379	–0.337723
H	–3.350280	–1.956235	1.275667
H	–4.625302	0.628500	–1.917217
S	4.285271	–3.236280	–0.714171
S	–5.576897	–1.779504	–0.410102

O	1.690979	4.362330	–0.919643
H	0.952879	3.719700	–0.869247
H	1.281578	5.233675	–0.891983
O	3.077046	3.773572	1.468066
H	3.842809	3.236679	1.240604
H	2.674825	4.027879	0.614558
C	–6.569672	–1.055761	–1.738036
H	–6.866032	–0.034513	–1.504035
H	–6.046561	–1.091797	–2.692393
H	–7.461480	–1.677567	–1.798971
C	4.415562	–4.392877	0.670763
H	4.824278	–3.909797	1.557097
H	3.456080	–4.854477	0.899780
H	5.108080	–5.164696	0.338360

B3LYP/def2-TZVP energy =
–1644.226106708019

TS with linkers (F_z = –0.05 V Å^{-1})

B3LYP/def2-TZVP geometry:

S	6.327650	–0.887979	–0.543193
S	–6.556763	–0.745752	–0.106088
C	3.953883	–1.327173	0.902030
C	2.587923	–1.159627	1.084646
C	1.794357	–0.422354	0.193072
C	2.456380	0.144084	–0.911951
C	3.819260	–0.021588	–1.101952
C	4.594548	–0.753558	–0.196601
H	4.507332	–1.908768	1.625696
H	2.120893	–1.620466	1.948049
H	1.886633	0.700821	–1.642083
H	4.289814	0.422339	–1.972076
C	0.347783	–0.308494	0.453675
H	–0.049614	–1.121152	1.050012
C	–0.585715	0.394298	–0.336734
O	–0.289091	1.392739	–1.075293
C	–2.039804	0.045269	–0.245983
C	–2.510907	–1.201203	0.172361
C	–2.989729	1.002970	–0.626265
C	–3.870175	–1.482831	0.230043
H	–1.813808	–1.984584	0.437733
C	–4.344433	0.736920	–0.566780
H	–2.640535	1.966608	–0.970942
C	–4.806828	–0.513709	–0.134052
H	–4.186113	–2.464436	0.552485
H	–5.056104	1.501297	–0.855451
O	1.077842	3.120540	0.148431
H	0.597332	2.444114	–0.475809
H	2.011927	3.132983	–0.094127
H	0.877016	2.498575	1.314622
O	0.638434	1.788195	2.139824
H	0.407198	0.872058	1.561138
H	–0.147971	2.072094	2.627164
C	6.986793	–1.805333	0.870007
H	6.820587	–1.271714	1.804932
H	6.567032	–2.808658	0.929602
H	8.058500	–1.881684	0.692439
C	–6.773997	–2.409043	0.571846
H	–6.339945	–3.167342	–0.078144
H	–6.358563	–2.486789	1.575445
H	–7.851048	–2.561021	0.622364

B3LYP/def2-TZVP energy =
–1644.177611808414

Enol-form with linkers (F_z = –0.05 V Å^{-1}) - final

B3LYP/def2-TZVP geometry:

C	4.092980	–1.249264	–0.284781
C	2.706325	–1.293685	–0.228721
C	1.905967	–0.230872	–0.671403
C	2.576885	0.882080	–1.212111
C	3.959040	0.934218	–1.272958
C	4.742930	–0.128009	–0.802397
H	4.655921	–2.097375	0.078146
H	2.227735	–2.178418	0.174710
H	2.007990	1.711843	–1.609896
H	4.439210	1.805749	–1.702507
C	0.452505	–0.357493	–0.605568
H	1.787778	2.655790	2.205127
H	0.084469	–1.375335	–0.592810

C	–0.487889	0.610621	–0.508232
O	–0.229961	1.933981	–0.328915
C	–1.933565	0.319747	–0.556846
C	–2.447475	–0.794273	–1.238951
C	–2.847255	1.160396	0.084598
C	–3.802718	–1.072845	–1.242030
H	–1.779369	–1.438402	–1.795441
C	–4.209919	0.889234	0.084734
H	–2.481288	2.037312	0.599940
C	–4.707957	–0.235974	–0.572901
H	–4.169005	–1.937301	–1.782817
H	–4.874264	1.564670	0.604301
O	1.932110	2.704437	1.252848
H	0.607977	2.092468	0.154987
H	2.731240	2.191347	1.076812
O	–1.808618	–3.420834	0.820805
H	–2.299005	–2.667502	0.467603
H	–1.720876	–3.240240	1.763198
S	–6.413030	–0.689909	–0.647791
S	6.497552	0.056803	–0.928545
C	–7.251988	0.582993	0.326260
H	–8.307165	0.315074	0.302455
H	–6.911125	0.583208	1.360514
H	–7.127380	1.571196	–0.114108
C	7.154121	–1.450947	–0.174548
H	8.237240	–1.349141	–0.222032
H	6.853084	–1.539360	0.868411
H	6.857929	–2.339812	–0.729909

B3LYP/def2-TZVP energy =
–1644.2072595081725

Keto-form with linkers (F_z = +0.05 V Å^{-1})

B3LYP/def2-TZVP geometry:

C	2.432908	–2.047452	0.975858
C	1.547709	–1.042258	1.355456
C	1.477220	0.165076	0.667877
C	2.328889	0.343494	–0.427633
C	3.210506	–0.649806	–0.816967
C	3.274657	–1.863313	–0.119943
H	2.454513	–2.966321	1.544107
H	0.908560	–1.210500	2.214506
H	2.304627	1.272244	–0.983988
H	3.857493	–0.485004	–1.670085
C	0.518582	1.263684	1.093404
H	1.064581	2.196356	1.244943
H	0.055952	0.997826	2.042524
C	–0.537368	1.527448	0.027489
O	–0.336929	2.391795	–0.819932
C	–1.783522	0.727276	–0.013003
C	–2.135070	–0.201785	0.976111
C	–2.663754	0.906236	–1.087028
C	–3.314854	–0.915809	0.896072
H	–1.487477	–0.373714	1.823305
C	–3.844955	0.193065	–1.179125
H	–2.401751	1.617976	–1.857799
C	–4.189443	–0.730327	–0.184339
H	–3.566132	–1.626815	1.673302
H	–4.492648	0.358939	–2.027143
S	4.442536	–3.054860	–0.707435
S	–5.660153	–1.692536	–0.181848
O	1.878218	4.058829	–1.002449
H	1.064809	3.512936	–0.971230
H	1.664318	4.835025	–1.530119
O	2.891899	4.120350	1.611185
H	3.696681	3.596899	1.539988
H	2.562341	4.195037	0.693425
C	–6.513539	–1.219302	–1.705868
H	–6.779441	–0.163623	–1.703693
H	–5.920675	–1.462651	–2.585821
H	–7.425408	–1.814191	–1.718243
C	4.200601	–4.478896	0.380667
H	4.434525	–4.237603	1.416901
H	3.188187	–4.873374	0.303242
H	4.901455	–5.233772	0.027325

B3LYP/def2-TZVP energy =
–1644.2267219080138

TS with linkers (F_z = +0.05 V Å^{-1})

B3LYP/def2-TZVP geometry:

S	6.288932	–0.923714	–0.535186
S	–6.545053	–0.798899	–0.020884
C	3.947876	–1.236327	0.994702
C	2.588978	–1.036874	1.200024
C	1.782039	–0.354619	0.278312
C	2.420441	0.120371	–0.881433
C	3.774847	–0.079431	–1.095990
C	4.565493	–0.753859	–0.159430
H	4.514122	–1.772015	1.743769
H	2.137834	–1.429885	2.104685
H	1.838551	0.624493	–1.639841
H	4.224000	0.283735	–2.013282
C	0.340884	–0.205941	0.563222
H	–0.057565	–0.992106	1.194153
C	–0.601579	0.451804	–0.259629
O	–0.320257	1.425153	–1.033950
C	–2.048161	0.083803	–0.159824
C	–2.490067	–1.184688	0.220664
C	–3.017501	1.034888	–0.505770
C	–3.843173	–1.496457	0.272054
H	–1.772786	–1.960811	0.452473
C	–4.365941	0.739865	–0.447595
H	–2.690408	2.012930	–0.831349
C	–4.800198	–0.534357	–0.055264
H	–4.138379	–2.495967	0.556925
H	–5.094118	1.494838	–0.718863
O	1.146401	3.149734	0.023652
H	0.614583	2.447978	–0.545352
H	2.071867	3.114728	–0.248660
H	0.957480	2.626893	1.197470
O	0.687574	1.981817	2.097697
H	0.436123	1.021367	1.574474
H	–0.112358	2.320746	2.522725
C	6.924147	–1.966347	0.799358
H	6.845214	–1.475771	1.768949
H	6.419498	–2.931540	0.823934
H	7.976852	–2.123220	0.568637
C	–6.721102	–2.553468	0.383900
H	–6.247641	–3.183412	–0.367763
H	–6.326092	–2.781570	1.372956
H	–7.793385	–2.743223	0.379421

B3LYP/def2-TZVP energy =
–1644.1737432084453

Enol-form with linkers (F_z = +0.05 V Å^{-1})

B3LYP/def2-TZVP geometry:

C	3.900553	–1.240526	1.038748
C	2.516574	–1.169437	1.122673
C	1.748199	–0.432097	0.210579
C	2.449460	0.212095	–0.827165
C	3.828948	0.145456	–0.919198
C	4.581364	–0.575481	0.018143
H	4.437465	–1.823068	1.773810
H	2.014342	–1.704535	1.920269
H	1.906953	0.748149	–1.594341
H	4.329850	0.639227	–1.743539
C	0.292244	–0.434582	0.340150
H	1.830862	3.421851	1.213427
H	–0.113143	–1.266475	0.901757
C	–0.612770	0.481418	–0.088786
O	–0.291259	1.678910	–0.640303
C	–2.071015	0.281323	0.015499
C	–2.647971	–0.994442	0.114714
C	–2.933479	1.381807	0.004066
C	–4.015730	–1.154692	0.226502
H	–2.025456	–1.877578	0.064342
C	–4.309196	1.228327	0.116107
H	–2.517553	2.374742	–0.090829
C	–4.870369	–0.043490	0.234426
H	–4.432186	–2.152943	0.286317
H	–4.933780	2.110013	0.107677
S	6.336124	–0.574084	–0.196116
S	–6.598755	–0.372785	0.387210
O	1.936107	3.057387	0.327104
H	0.564650	2.027815	–0.313335
H	2.710416	2.480414	0.365848
O	–0.243117	–2.966155	–1.810135
H	–0.766731	–2.757185	–2.591637
H	0.010169	–2.107950	–1.443803
C	6.932538	–1.719577	1.070517

H	6.716138	–1.359188	2.075460
H	6.519379	–2.717430	0.929232
H	8.012329	–1.761225	0.935717
C	–7.376671	1.249696	0.196938
H	–7.098439	1.927545	1.002728
H	–7.138662	1.692411	–0.769148
H	–8.448347	1.063091	0.246408

B3LYP/def2-TZVP energy = –1644.2049384081913

c) Molecular junctions

Off-state

B3LYP/Lanl2MB geometry:

S	6.17278200	3.63798200	–1.80305400
S	–6.23032700	3.70014900	1.78188500
C	3.50081400	4.35312800	–2.29257300
C	2.13719300	4.22263400	–2.04720200
C	1.64916400	3.32500700	–1.10333500
C	2.57587200	2.55040300	–0.40218700
C	3.93522400	2.66922500	–0.63475000
C	4.41863100	3.57582200	–1.58650400
H	3.83267200	5.06192200	–3.03756900
H	1.44480100	4.83818700	–2.61061500
H	2.23030000	1.84016400	0.34029700
H	4.63109400	2.05449600	–0.07665500
C	0.17467600	3.19412500	–0.83894400
H	–0.08349900	2.15076900	–0.63323600
H	–0.39776400	3.47115200	–1.72928900
C	–0.34521600	4.03988900	0.32638400
O	0.39122600	4.76930000	0.95888500
C	–1.79790200	3.94479300	0.66828400
C	–2.69960100	3.12132200	–0.01697000
C	–2.28551700	4.71467100	1.72720000
C	–4.03412800	3.07061300	0.34453600
H	–2.36709200	2.50799000	–0.84297000
C	–3.61936200	4.67199000	2.09760200
H	–1.59384900	5.35288900	2.26051000
C	–4.51300200	3.84580900	1.40848800
H	–4.71328600	2.42553100	–0.19926300
H	–3.95425300	5.28374600	2.92259600
C	6.65392900	5.21062400	–1.05022300
H	6.19887700	6.05681300	–1.56331900
H	6.41224200	5.23587300	0.01147100
H	7.73502000	5.26713400	–1.16777100
C	–6.95110100	5.24073000	1.16473200
H	–6.53957500	6.10813100	1.67854000
H	–6.81994400	5.34022400	0.08833700
H	–8.01478600	5.16923700	1.38680400
Au	–7.28296400	2.09718000	0.25710900
Au	–8.52035000	0.21287200	–1.53528100
Au	–10.10708700	1.56485500	0.45188000
Au	–8.20441800	–0.44406100	1.25093600
Au	–11.34399700	–0.31962400	–1.34066200
Au	–11.02806400	–0.97655800	1.44555600
Au	–9.44132700	–2.32854100	–0.54160500
Au	–9.75726000	–1.67160700	–3.32782200
Au	–12.93073400	1.03235800	0.64650000
Au	–9.12539100	–2.98547900	2.24461900
Au	7.26331300	2.07780700	–0.26062200
Au	8.54524400	0.24380600	1.55252300
Au	10.08941700	1.56852100	–0.48587400
Au	8.19523900	–0.47497900	–1.21428500
Au	11.37087600	–0.26565300	1.32743300
Au	11.02087100	–0.98443800	–1.43937600
Au	9.47669700	–2.30915300	0.59902100
Au	9.82670300	–1.59036700	3.36582900
Au	12.91504900	1.05906100	–0.71096400
Au	9.12668800	–3.02794300	–2.16779400

B3LYP/Lanl2MB energy = –3415.6177382

On-state

B3LYP/Lanl2MB geometry:

S	6.24746700	–2.99515200	0.58763000
S	–5.60074100	1.87479100	0.01496200
C	3.48928700	–3.17533000	0.91768900
C	2.22777400	–2.65027200	0.78703600
C	2.01167500	–1.36025000	0.22691400
C	3.16966800	–0.61962100	–0.14662300

C	4.42644800	–1.14252600	–0.03224200
C	4.61872000	–2.44362700	0.49459600
H	3.60680600	–4.14962800	1.36790800
H	1.40279900	–3.21772800	1.19643100
H	3.04260400	0.37659100	–0.55049300
H	5.28588800	–0.56179900	–0.34202300
C	0.74524700	–0.74342200	0.05711500
H	0.77084100	0.31947500	–0.13488700
C	–0.51891600	–1.32172900	0.03257600
C	–1.74534700	–0.55862900	0.02049300
C	–1.77837400	0.81615400	0.34216500
C	–2.96417600	–1.18907200	–0.29726000
C	–2.95875600	1.51692100	0.33225100
H	–0.87425700	1.33415000	0.62890900
C	–4.15140700	–0.48845500	–0.31181800
H	–2.96762600	–2.23991600	–0.54674200
C	–4.17172000	0.87941400	0.00101600
H	–2.95993000	2.56812600	0.59019000
H	–5.06142800	–1.00840800	–0.57102900
C	6.40896100	–4.26099200	–0.69157200
H	5.75334900	–5.10580300	–0.48975200
H	6.22164800	–3.84429600	–1.67911400
H	7.44503500	–4.58869800	–0.63154400
C	–5.95480800	2.15169100	1.76705100
H	–6.17722700	1.21675200	2.27806500
H	–5.13597800	2.67480900	2.25723400
H	–6.84036800	2.78453100	1.78043700
O	–0.70345600	–2.64955000	–0.01859400
H	0.11245100	–3.11051700	–0.25750700
Au	–7.55826500	0.53929800	–0.60717900
Au	–9.85935400	–1.03058600	–1.33851300
Au	–10.16565500	1.76017000	–0.69643600
Au	–9.53253500	–0.13383100	1.37872800
Au	–12.46646200	0.18984200	–1.42771600
Au	–12.13964200	1.08659800	1.28952600
Au	–11.83334200	–1.70415800	0.64744900
Au	–12.16016100	–2.60091400	–2.06979200
Au	–12.77276200	2.98059800	–0.78563900
Au	–11.50652100	–0.80740400	3.36470000
Au	7.76131500	–1.28967700	–0.30798300
Au	9.54085800	0.71512600	–1.36078500
Au	10.59240400	–1.80354800	–0.44158400
Au	9.56297600	0.10495700	1.45374600
Au	12.37155900	0.20161300	–1.49436100
Au	12.39367800	–0.40855600	1.32017000
Au	11.34213200	2.11011800	0.40096900
Au	11.32001400	2.72028700	–2.41356300
Au	13.42310500	–2.31706100	–0.57515900
Au	11.36424900	1.49995100	3.21550900

B3LYP/Lanl2MB energy = –3415.4055336

Supplementary References

1. Bai, J. *et al.* Anti-resonance features of destructive quantum interference in single-molecule thiophene junctions achieved by electrochemical gating. *Nat. Mater.* **18**, 364–369 (2019).
2. Capozzi, B. *et al.* Single-molecule diodes with high rectification ratios through environmental control. *Nat. Nanotechnol.* **10**, 522–527 (2015).
3. Stevenson, D. The Strengths of chemical bonds. *J. Am. Chem. Soc.* **77**, 2350–2350 (1955).
4. Stuyver, T., De Proft, F., Geerlings, P. & Shaik, S. How Do Local Reactivity Descriptors Shape the Potential Energy Surface Associated with Chemical Reactions? The Valence Bond Delocalization Perspective. *J. Am. Chem. Soc.* **142**, 10102–10113 (2020).
5. Shaik, S. S. & Hiberty, P. C. *A chemist's guide to valence bond theory*. (John Wiley & Sons, 2007).
6. Ioffe, A. & Shaik, S. Intramolecular effects in the cycloaddition of three ethylenes vs. the Diels–Alder reaction. *J. Chem. Soc., Perkin trans. 2*, 2101–2108 (1992).
7. Migliore, A., Schiff, P. & Nitzan, A. On the relationship between molecular state and single electron pictures in simple electrochemical junctions. *Phys. Chem. Chem. Phys.* **14**, 13746–13753 (2012).
8. Sedghi, G. *et al.* Long-range electron tunnelling in oligo-porphyrin molecular wires. *Nat. Nanotechnol.* **6**, 517–523 (2011).
9. Hong, W. *et al.* Trimethylsilyl-terminated oligo (phenylene ethynylene) s: an approach to single-molecule junctions with covalent Au–C σ-bonds. *J. Am. Chem. Soc.* **134**, 19425–19431 (2012).
